# Grp94 Regulates the Recruitment of Aneural AChR Clusters for the Assembly of Postsynaptic Specializations by Modulating ADF/Cofilin Activity and Turnover

**DOI:** 10.1523/ENEURO.0025-20.2020

**Published:** 2020-09-02

**Authors:** Zora Chui-Kuen Chan, Linyan Deng, Chi Wai Lee

**Affiliations:** School of Biomedical Sciences, Li Ka Shing Faculty of Medicine, The University of Hong Kong, Hong Kong

**Keywords:** acetylcholine receptor, ADF/cofilin, Grp94, heat shock protein, neuromuscular junction, temperature stress

## Abstract

Temperature is a physiological factor that affects neuronal growth and synaptic homeostasis at the invertebrate neuromuscular junctions (NMJs); however, whether temperature stress could also regulate the structure and function of the vertebrate NMJs remains unclear. In this study, we use *Xenopus laevis* primary cultures as a vertebrate model system for investigating the involvement of heat shock protein 90 (HSP90) family of stress proteins in NMJ development. First, cold temperature treatment or HSP90 inhibition attenuates the formation of aneural acetylcholine receptor (AChR) clusters, but increases their stability after they are formed, in cultured muscles. HSP90 inhibition specifically affects the stability of aneural AChR clusters and their associated intracellular scaffolding protein rapsyn, instead of causing a global change in cell metabolism and protein expression in *Xenopus* muscle cultures. Upon synaptogenic stimulation, a specific HSP90 family member, glucose-regulated protein 94 (Grp94), modulates the phosphorylation and dynamic turnover of actin depolymerizing factor (ADF)/cofilin at aneural AChR clusters, leading to the recruitment of AChR molecules from aneural clusters to the assembly of agrin-induced postsynaptic specializations. Finally, postsynaptic Grp94 knock-down significantly inhibits nerve-induced AChR clustering and postsynaptic activity in nerve-muscle co-cultures as demonstrated by live-cell imaging and electrophysiological recording, respectively. Collectively, this study suggests that temperature-dependent alteration in Grp94 expression and activity inhibits the assembly of postsynaptic specializations through modulating ADF/cofilin phosphorylation and activity at aneural AChR clusters, which prevents AChR molecules from being recruited to the postsynaptic sites via actin-dependent vesicular trafficking, at developing vertebrate NMJs.

## Significance Statement

Heat shock protein 90 (HSP90) is one of the most studied and abundant molecular chaperones of eukaryotic cells that protect proteins from cellular stress. Our study provides the first evidence showing that temperature-dependent alteration in the expression and activity of a specific HSP90 family member glucose-regulated protein 94 (Grp94) regulates the recruitment of aneural acetylcholine receptor (AChR) clusters for the assembly of postsynaptic specializations through actin depolymerizing factor (ADF)/cofilin-mediated vesicular trafficking at developing vertebrate neuromuscular junctions (NMJs). Given the recent identification of Grp94 and other endoplasmic reticulum (ER) chaperones as potential biomarkers for diagnosis of myasthenia gravis, an autoimmune NMJ disease, results of this study not only enhance our understanding on the fundamental mechanisms underlying NMJ development but also provide insights into the pathogenic mechanisms underlying ER stress response and NMJ disruption in neuromuscular diseases.

## Introduction

Synapses are the fundamental structures in the nervous system that enable efficient communication between neurons and their target cells. Neuromuscular junction (NMJ), a peripheral synapse, is formed between a motor neuron and a skeletal muscle fiber. Because of its accessibility and simplicity in structure, NMJ has served as a model synapse for elucidating the molecular mechanisms underlying synapse formation and maintenance in health, disease, and aging (Sanes and Lichtman, 2001; Li et al., 2018; Chan et al., 2020a). At developing NMJs, aggregation of acetylcholine receptors (AChRs) at the postsynaptic membranes represents an important step in neuromuscular synaptogenesis. Before nerve innervation, AChR molecules are both diffusely distributed throughout the muscle surface and spontaneously clustered in the form of AChR prepatterns (Yang et al., 2000, 2001; Lin et al., 2001). Upon synaptogenic induction, nerve-induced AChR clustering at the postsynaptic sites is believed to be contributed by the recruitment of both diffuse and prepatterned AChRs, as well as the local synthesis of AChR proteins at the sub-synaptic nuclei (Sanes and Lichtman, 2001). The postsynaptic specializations at NMJs are associated with dense networks of stable filamentous actin (F-actin) structures at the cell cortex, which mediate AChR cluster formation and redistribution through rapsyn (Dai et al., 2000; Borges and Ferns, 2001; Dobbins et al., 2008). A previous study has demonstrated that actin depolymerizing factor (ADF)/cofilin-mediated actin dynamics regulate the vesicular trafficking of AChRs at developing NMJs (Lee et al., 2009). These findings suggest a novel ADF/cofilin-dependent transcytosis mechanism underlying the redistribution of aneural AChR clusters for the assembly of synaptic AChR clusters at NMJs. However, the mechanistic regulation of AChR redistribution from aneural to synaptic clusters at developing NMJs remains unclear.

Heat shock proteins (HSPs) are molecular chaperones that show remarkable sequence homology across the phylogenetic spectrum from a unicellular organism, *Saccharomyces cerevisiae*, to a multicellular organism, mammal. These stress proteins are grouped into major families according to their approximate molecular weight in kDa. Among them, HSP90 is a highly abundant and ubiquitous molecular chaperone, which plays an essential role in many different processes to maintain cellular homeostasis under stressful conditions (Schopf et al., 2017). The HSP90 family includes cytosolic HSPs (HSP90α and HSP90β), endoplasmic reticulum (ER)-resident glucose-regulated protein 94 (Grp94), and mitochondrial-specific tumor necrosis factor receptor-associated protein-1 (TRAP-1). Grp94, encoded by the HSP90B1 gene, shares many biochemical features with other HSP90 proteins (Csermely et al., 1998; Marzec et al., 2012). It is believed that Grp94 can escape ER retention and retrieval in cells under ER stress (Gutiérrez and Simmen, 2014). Intriguingly, a previous study suggested that Grp94 can also be found in the cell surface of C2C12 myotubes, in which Grp94 phosphorylation mediated by the Src kinase Fyn promotes the chaperone export from the ER during the early phase of myoblast differentiation (Frasson et al., 2009). Given that the cytosolic HSP90β is known to regulate AChR cluster formation and maintenance through modulating rapsyn turnover (Luo et al., 2008), whether the ER-resident HSP90 family member, Grp94, is involved in NMJ development remains unknown.

In this study, we first show that temperature stress up-regulates the mRNA transcript levels of both HSP90β and Grp94, but down-regulates the protein level of only Grp94, in cultured *Xenopus* muscle cells. Interestingly, pharmacological inhibition of HSP90 activity by 17-(allylamino)−17-demethoxygeldanamycin (17-AAG) or molecular manipulation of endogenous Grp94 expression suppresses the formation of aneural AChR clusters and increases the stability of aneural AChR clusters after they are formed. Upon synaptogenic stimulation, the recruitment of preexisting AChRs to agrin-induced AChR clusters is significantly reduced in wild-type (WT) muscle cells treated with 17-AAG and in Grp94 knock-down muscle cells. Interestingly, 17-AAG treatment accelerates the turnover of green fluorescent protein-tagged *Xenopus* ADF/cofilin (GFP-XAC) at both perforated and AChR-rich regions of aneural clusters resembling the dynamic turnover of GFP-XAC inactive (S3E) mutant, suggesting that HSP90 regulates dephosphorylation and activation of ADF/cofilin. Finally, nerve-induced AChR clustering and synaptic functions are impaired in chimeric nerve-muscle co-cultures containing Grp94 knock-down muscles and WT neurons, indicating the essential roles of postsynaptic Grp94 in regulating synaptic structure and function of developing NMJs. Together, our study suggests that temperature-dependent alteration in Grp94 expression and activity regulates the recruitment of AChR molecules from aneural to agrin-induced synaptic clusters through modulating ADF/cofilin phosphoregulation to mediate actin-dependent vesicular trafficking at developing NMJs.

## Materials and Methods

### Embryo microinjection and primary culture from *Xenopus* embryos

Adult female frogs (*Xenopus* 1, RRID: XEP_Xla100) were injected with 1000 I.U. human chorionic gonadotropin (hCG; Sigma, catalog #CG10) with 0.1% BSA to induce ovulation. After fertilization *in vitro*, embryos were maintained in Holtfreter’s solution (v/v; 60 mm NaCl, 0.6 mm KCl, 0.9 mm CaCl_2_, and 0.2 mm NaHCO_3_; pH 7.4). A total of 20–100 pg of DNA constructs encoding either WT or phosphorylation mutant forms (S3A and S3E) of GFP-XAC (gifts from Dr. James Bamburg, Colorado State University) were microinjected into one blastomere of one- or two-cell stage *Xenopus* embryos with an oocyte injector. GFP-expressing embryos were screened for primary culture preparation. Myotomal muscle tissues and neural tubes were isolated from stage 19–22 *Xenopus* embryos after enzymatic digestion with 1 mg/ml collagenase (Sigma-Aldrich, catalog #C98191G), followed by dissociation with calcium-magnesium-free solution. Dissociated muscle cells were then plated on glass bottom dishes or glass coverslips coated with entactin-collagen IV-laminin (ECL) cell attachment matrix (Merck Millipore, catalog #08-100). The ECL coating was performed by incubating the coverslips or dishes with 10 μg/ml ECL in 10% Leibovitz’s L-15 medium (Sigma-Aldrich, catalog #L4386) at 37°C for 3 h. The coating was later washed with PBS solution (137 mm NaCl, 2.7 mm KCl, 10 mm Na_2_HPO_4_, 1.8 mm KH_2_PO_4_), followed by Steinberg’s solution [60 mm NaCl, 0.67 mm KCl, 0.35 mm Ca(NO_3_)_2_, 0.83 mm MgSO_4_, and 10 mm HEPES; pH 7.4]. Cells were cultured in medium containing 87% (v/v) Steinberg’s solution, 10% (v/v) Leibovitz’s L-15 medium, 1% fetal bovine serum (Invitrogen, catalog #10270), 1% penicillin/streptomycin (Thermo Fisher Scientific, catalog #15140122), and 1% gentamicin sulfate (Thermo Fisher Scientific, catalog #15750060). Muscle cells were kept at 22°C for at least 24 h to allow cell attachment and aneural AChR cluster formation before treatments, if any. To make nerve-muscle or bead-muscle coculture, dissociated spinal neurons or polystyrene latex beads coated with agrin (R&D Systems, catalog #550-AG-100/CF) were added in 2-d-old muscle cultures and maintained for 1 d before imaging. All the experiments involving *Xenopus* frogs and embryos were performed in accordance with The University of Hong Kong animal care committee’s regulations.

### Morpholino-mediated knock-down of endogenous proteins

Knock-down of endogenous proteins in *Xenopus* was achieved by embryonic injection of antisense morpholino oligonucleotides (MO; Gene Tools), which bind to the target mRNA sequence that block its protein translation. The following MO sequences were used in this study: *Xenopus* Grp94 MO: 5′-GACCGATTGCCCAAAACTTCCTCAT-3′, *Xenopus* HSP90β MO: 5′-CATTGTGGGCAACTTCTGGCATC-3′, and Control MO: 5′-CCTCT TACCT CAGTT ACAAT TTATA-3′. To visualize the presence of MO in the microinjected embryos, MOs were co-injected with Alexa Fluor 488-conjugated dextran (Thermo Fisher Scientific, catalog #D22910) as a cell lineage tracer. The effectiveness of MO-mediated knock-down of endogenous proteins was validated by Western blot analyses.

### Quantitative real-time RT-PCR analysis of HSP90 expression

Total RNA was extracted using the TRIzol reagent from 2-d-old *Xenopus* muscle cultures incubated at 22°C, 15°C, or 10°C, respectively. Isolated RNA samples were treated with DNase (Thermo Fisher Scientific, catalog #EN0521) to remove genomic DNA. An equal amount of RNA from samples was reverse transcribed into cDNA with High-Capacity cDNA Reverse Transcription kit (Thermo Fisher Scientific, catalog #4368814) and qPCR was performed using CXF96 Touch together with SYBR Premix Ex Taq (Takara Bio, catalog #RR420A). Data were acquired and analyzed with CFX Manager (Bio-Rad, RRID: SCR_017251). Primers are listed below:

HSP90α forward: 5′-TCTGACTGACCCAAGCAAAC-3′;

HSP90α reverse: 5′-GCCTGCAAAGCCTCCATAAA-3′;

HSP90β forward: 5′-CTATGATTGATACCGGAATT-3′;

HSP90β reverse: 5′-CATATTGCTCATCATCATTG-3′;

Grp94 forward: 5′-CACTGATGACCCTCGTGGTG-3′;

Grp94 reverse: 5′-AGGGGCTCCTCTACTGTCTC-3′;

TRAP-1 forward: 5′-CCCAGGGACAAAGGTTGTGA-3′;

TRAP-1 reverse: 5′-TCATGCTGCCATTCCCCAAT-3′;

GAPDH forward: 5′-GTGTATGTGGTGGAATCT-3′;

GAPDH reverse: 5′-AAGTTGTCGTTGATGACCTTTGC-3′.

### Pharmacological treatment

For experiments studying the effect of HSP90 on aneural AChR cluster formation, muscle cultures were pretreated with different concentrations (0.1 nm, 0.25 nm, or 1 nm) of 17-AAG (ApexBio, catalog #A4054-10). To investigate the effect of HSP90 in the remodeling of AChR clusters, 2-d-old muscle cultures were treated with 17-AAG (1 nm) or PU-WS13 (15 μm; ApexBio, catalog #B5885). For experiments investigating the contribution of aneural AChR clusters to agrin-induced synaptic AChR clusters, 2-d-old muscle cells were treated with 17-AAG (1 nm) from 1 h before photobleaching experiments. For the experiments investigating the nerve-induced AChR clusters, PU-WS13 (15 μm) was applied to 2-d-old muscle cultures from 1 h before spinal neurons plating. For experiment investigating inhibition of HSP90 activity on rapsyn localization in aneural AChR clusters, 2-d-old muscle cultures were treated with 1 nm 17-AAG from 1 h before agrin bead addition. For experiments investigating the HSP90 and Grp94 activity in rapsyn localization and AChR internalization, 2-d-old muscle cultures were treated with 1 nm 17-AAG or 15 μm PU-WS13 from 1 h before plating spinal neurons or adding agrin beads.

### Labeling of different AChR pools in cultured muscle cells

To differentially identify the preexisting and newly synthesized AChRs, surface AChRs in 2-d-old muscle cultures were first labeled with 0.1 μm tetramethylrhodamine (Rh)-conjugated (Thermo Fisher Scientific, catalog #T1175), Alexa Fluor 488-conjugated (488; Thermo Fisher Scientific, catalog #B13422), or Alexa Fluor 647-conjugated (647; Thermo Fisher Scientific, catalog #B35450) α-bungarotoxin (BTX) for 45 min, followed by extensive washing with culture medium. Cells were then saturated with a high dose of unconjugated BTX (6 μm, Thermo Fisher Scientific, catalog #B1601) for 30 min, followed by extensive washing with culture medium. Newly synthesized and inserted AChRs were then labeled with 1 μm 488-BTX or 647-BTX at different timepoints, as specified. For live imaging, glass coverslips with culture cells were mounted on sealed live chamber containing culture medium. To determine the density of surface AChRs, cultured muscle cells were first labeled with 0.2 μm biotin-XX-conjugated BTX (biotin-BTX; Thermo Fisher Scientific, catalog #B1196) for 45 min, then fixed with 4% paraformaldehyde in PBS for 15 min and permeabilized with 0.5% Triton X-100 in PBS for 10 min. The samples were then incubated with 0.16 μm Qdot 655 (QD)-conjugated streptavidin (Thermo Fisher Scientific, catalog #Q10143MP) for 10 min. For experiments testing the possibility of AChR photo-dissipation, surface AChRs were labeled with 1 μm Alexa Fluor 594-BTX (594-BTX; Thermo Fisher Scientific, catalog #B13423) or rhodamine-BTX (Rh-BTX) for 45 min, followed by extensive washing with culture medium. After 6 or 24 h, newly inserted surface AChRs were labeled with 1 μm 488-BTX for 45 min. To differentially identify surface and internal AChRs, cells were first fixed with 4% paraformaldehyde in PBS for 15 min. Surface AChRs were labeled with 0.1 μm Rh-BTX for 45 min, followed by saturation with 6 μm unconjugated BTX for 30 min. After cell permeabilization with 0.5% Triton X-100, internal AChRs were labeled with 0.1 μm 488-BTX for 45 min. Coverslips were then mounted on glass slides with the anti-bleaching reagent fluoromount-G (Thermo Fisher Scientific, catalog #00-4958-02) for later observation.

### Fixation and immunostaining of cells

*Xenopus* muscle cultures were fixed with 4% paraformaldehyde in PBS for 15 min. After extensive washing with PBS, fixed cells were permeabilized with 0.5% Triton X-100 in PBS, followed by blocking with 2% BSA at room temperature for 2 h or at 4°C overnight. Cells were incubated with primary antibodies, including XAC (1:500, a gift from Dr. James Bamburg, Colorado State University), p34-Arc/ARPC2 (1:100; EMD Millipore, catalog #07-227, RRID: AB_310447), rapsyn (1:100; Affinity Bioreagent, catalog #MA1-746, RRID: AB_2177611), or vinculin (1:250; Sigma-Aldrich, catalog #V4505, RRID: AB_477617) for 2 h, followed by fluorophore-conjugated secondary antibodies (Thermo Fisher Scientific, catalog #A-11029, RRID: AB_2534088; catalog #A-21206, RRID: AB_2535792) for 45 min. Coverslips were then mounted on glass slides with the antibleaching reagent fluoromount-G for later observation.

### Quantitative metabolomics

#### Extraction and derivatization of polar metabolites

A total of 1000 μl methanol/water (80%, v/v) with 200 ng norvaline internal standard was added to the samples. The samples were homogenized after two cycles of sonication at 10 μm for 20 s on ice and 10-s pause time. The samples were centrifuged for 5 min at 16,000 × *g* at 4°C. A total of 500 μl supernatant was dried under a gentle stream of nitrogen at room temperature for derivatization. The dried residue was redissolved and derivatized for 2 h at 37°C in 40 μl methoxylamine hydrochloride (30 mg/ml in pyridine), followed by trimethylsilylation for 1 h at 37°C in 70 μl MSTFA with 1% TMCS. Up to 1 μl sample was injected for gas chromatography-mass spectrometry (GC-MS)/MS analysis.

#### Extraction and transesterification of fatty acid metabolites

A total of 100 μl chloroform with 20 μg C19:0 fatty acid internal standard was spiked to the samples. The samples were homogenized after two cycles of sonication at 10 μm for 20 s on ice and 10-s pause time. The sample was centrifuged for 5 min at 16,000 × *g* at 4°C. The pellet was separated and stored at −80°C; 1 ml methanol and 50 μl concentrated hydrochloric acid (35%, w/w) were added to the sample. The solution was overlaid with nitrogen and the tube was tightly closed. After vortexing, the tube was heated at 100°C for 1 h. Once cooled to room temperature, 1 ml hexane and 1 ml water were added for FAMEs extraction. The tube was vortexed and after phase separation, up to 1 μl the hexane phase was injected for GC-MS analysis.

#### Data acquisition

GC-MS chromatogram was acquired in SCAN and MRM mode in an Agilent 7890B GC-Agilent 7010 Triple Quadrapole Mass Spectrometer system. For polar metabolites, the samples were separated through an Agilent DB-5MS capillary column (30 × 0.25 mm ID, 0.25-μm film thickness) under constant flow at 1 ml min^−1^. Characteristic quantifier and qualifier transitions were monitored in MRM mode during the run. For fatty acid metabolites, the samples were separated through an Aligent DB-23 capillary column (60 × 0.25 mm ID, 0.15-μm film thickness) under constant pressure at 33.4 psi. Characteristic fragment ions (*m/z* 55, 67, 69, 74, 79, 81, 83, 87, 91, 93, 95, 96, 97, 115, 127, 143) were monitored in SIM mode throughout the run. Mass spectra from *m/z* 50–350 were acquired in SCAN mode. Principal component analyses (PCAs) of polar metabolites and fatty acids was conducted with the determined data peaks using MetaboAnalyst 4.0 (Chong et al., 2019).

### Protein synthesis assay

To determine the effects of HSP90 inhibition on protein synthesis in cultured *Xenopus* muscle cells, 1 nm 17-AAG was added to culture medium before cell plating. For the positive control, 4-d-old muscle cells were treated with 25 μm cycloheximide (CHX; ApexBio, catalog #A8244-1000) for 2 h before adding O-propargyl-puromycin (OPP) reagent (Thermo Fisher Scientific, catalog #C10456). After 30 min, cells were fixed with 4% paraformaldehyde in PBS for 15 min and permeabilized with 0.5% Triton X-100 in PBS, followed by OPP detection according to manufacturer’s instructions.

### Immunoblotting

To validate the effectiveness of MO-mediated knock-down of endogenous proteins, the dorsal parts from *Xenopus* embryos at Nieuwkoop and Faber stage 19–22 were homogenized in RIPA buffer (Thermo Fisher Scientific, catalog #89900) containing 1% protease inhibitor cocktail and 1% EDTA. For experiments investigating the effects of temperature on HSP90β and Grp94 expression, 2-d-old cultured muscle cells incubated at 22°C or 10°C were scraped and homogenized in RIPA buffer containing 1% protease inhibitor cocktail and 1% EDTA. Protein lysates were obtained from the supernatant after high-speed centrifugation (15,000 × *g*). The concentration of protein lysates was determined by BCA protein assay kit (Thermo Fisher Scientific, catalog #23227); 10–30 μg protein lysates, Pierce lane marker non-reducing sample buffer (25% v/v to sample buffer, Thermo Fisher Scientific, catalog #39001), and β-mercaptoethanol (5%; v/v to sample buffer, Bio-Rad, catalog #1610710) were loaded and separated on a 10% TGX Stain-Free polyacrylamide gel, followed by transferring onto polyvinylidene difluoride (PVDF) membranes. The blot was blocked by immersing in 5% non-fat milk containing TBST for 1 h at room temperature. The blots were probed for the following primary antibodies: Grp94 (1:1000; Thermo Fisher Scientific, catalog #36-2600, RRID: AB_2533246), HSP90β (1:1000; Thermo Fisher Scientific, catalog #37-9400, RRID: AB_2533349), or β-tubulin (1:1000; DSHB, catalog #EVII-s, RRID: AB_528499) at 4°C overnight, followed by HRP-conjugated secondary antibodies (1:5000; Thermo Fisher Scientific, catalog #G-21040, RRID: AB_2536527; catalog #G-21234, RRID: AB_2536530) at room temperature for 1 h. Signals were visualized using Pierce enhanced chemiluminescence substrate (Thermo Fisher Scientific, catalog #32106), and image acquisition was performed with Image Lab 6.0.1 (Bio-Rad) by ChemiDoc XRS+ System (Bio-Rad).

### Electrophysiological recordings

For cultures used for electrophysiological recordings, myotubes and spinal neurons were plated together on plain glass coverslips. Spontaneous synaptic currents (SSCs) were recorded from myoballs in 1-d-old nerve-muscle coculture by whole-cell patch clamp recording. The cultures were perfused with the recording solution, containing 140 mm NaCl, 5 mm KCl, 1 mm CaCl_2_, 1 mm MgCl_2_, and 10 mm HEPES; pH 7.4. Glass micropipettes (Sutter instrument) with 1–3 MΩ was filled with intrapipette solution containing 145 mm KCl, 1 mm NaCl, 1 mm MgCl_2_, 1 mm Mg-ATP, and 10 mm HEPES; pH 7.2. Muscle cells were voltage clamped at –70 mV. All data were obtained using the MultiClamp 700B amplifier (Molecular Devices). The signals were filtered at 2 kHz and sampled at 20 kHz using Digidata 1440A (Molecular Devices, RRID: SCR_018455). The frequency of SSCs was defined as the number of events per second. The frequency, amplitude, rise time, decay time, and inter-event intervals of SSCs were analyzed using MiniAnalysis Program v6.0.3 (Synaptosoft, RRID: SCR_002184).

### Fluorescence microscopy

The fluorescent images were acquired on inverted epifluorescence microscopes (IX81 or IX83; Olympus) with an oil immersion 60× NA 1.42 objective lens or with a 20× NA 0.5 objective lens. Images were captured by iXon EMCCD camera (Andor) using the cell^R software (Olympus) on IX81, or by ORCA-Flash4.0 LT+ digital CMOS camera (Hamamatsu) using MicroManager software v.1.4 (Open Imaging, RRID: SCR_016865) on IX83. Quantitative measurements of fluorescence images were performed by ImageJ software (National Institute of Health, RRID: SCR_003070).

Fluorescence images of QD-labeled single AChR molecules were acquired on a super-resolution structured illumination microscopy (SR-SIM Elyra S1; Carl Zeiss) with an oil immersion 60× NA 1.4 objective lens. Images were obtained by a sCMOS camera (PCO Edge) with cooling system using ZEN 2.3 software (Carl Zeiss, RRID: SCR_018163).

Photobleaching of aneural AChR clusters was performed on a confocal microscope (LSM 800; Carl Zeiss) using 20× NA 0.8 objective lens and Diode laser line (488 or 561 nm) with 100% laser intensity. Identical settings were applied in all photobleaching experiments. A fluorescence image was taken immediately after photobleaching to ensure the complete photobleaching of AChR signals.

Fluorescence recovery after photobleaching (FRAP) experiments were performed in total internal reflection fluorescence (TIRF) mode using Axio TIRF unit fitted on an inverted microscope equipped with oil immersion 100× NA 1.46 DIC objective lens. Photobleaching was performed by using Sapphire laser line (488 nm) with 50% laser intensity. Images were captured through MetaMorph (Molecular Devices, RRID: SCR_002368) by Evolve 512 EMCC camera (Photometrics). Identical settings were applied in all photobleaching experiments. To obtain the baseline of fluorescence intensity before photobleaching, 10 images at 1-s interval were taken in muscle cells expressing WT or mutant forms of GFP-XAC before photobleaching. After photobleaching, time-lapse images were taken at 1-s interval until the fluorescence intensity has reached the plateau level.

### Statistical analysis

All data from this study were collected from at least three replicates in independent experiments. Prism 7.0 (GraphPad) was used for statistical analyses. Detailed statistical results, including the exact *p* values, are provided in Table 1.

**Table 1:** Summary of statistical analyses

Figures	Comparison		Statistical test	*p* value	*F*, Dfn, Dfd
1*B*	1 d	22°C vs 15°C	Two-way ANOVA, Turkey'smultiple comparison test	0.0163	Interaction: 15.96, 6, 18; time point:71.17, 3, 18; temperature: 142.8, 2, 6
		22°C vs 10°C		0.0019	
	2 d	22°C vs 15°C		0.0001	
		22°C vs 10°C		0.0001	
	3 d	22°C vs 15°C		0.0001	
		22°C vs 10°C		0.0001	
	4 d	22°C vs 15°C		0.0001	
		22°C vs 10°C		0.0001	
1*C*	HSP90α	22°C vs 15°C	Two-way ANOVA, Turkey'smultiple comparison test	0.5097	Interaction: 11.22, 6, 24; Gene: 38.81,3, 24; temperature: 43.31, 2, 24
		22°C vs 10°C		0.159	
		15°C vs 10°C		0.0155	
	HSP90β	22°C vs 15°C		0.0489	
		22°C vs 10°C		0.0024	
		15°C vs 10°C		0.4152	
	Grp94	22°C vs 15°C		<0.0001	
		22°C vs 10°C		<0.0001	
		15°C vs 10°C		0.0012	
	TRAP-1	22°C vs 15°C		0.2602	
		22°C vs 10°C		0.1889	
		15°C vs 10°C		0.9793	
1*E*	HSP90β	22°C vs 10°C	Unpaired *t* test with Welch'scorrection	0.7421	4.39, 2, 2
	Grp94	22°C vs 10°C		0.0035	3.03, 2, 2
1*F*		Control vs 0.25 nm	One-way ANOVA, Turkey'smultiple comparison test	0.0067	75.47, 3, 12
		Control vs 0.5 nm		<0.0001	
		Control vs 1 nm		<0.0001	
1*H*	Normalized AChR intensity cluster region	Control vs 17-AAG	Unpaired *t* test	0.0403	4.762, 2, 2
	AChR-poor perforations/AChR cluster area		Unpaired *t* test	0.02	2.462, 3, 3
1*K*	24 h	Control vs 17-AAG	Two-way ANOVA, Sidak'smultiple comparison test	0.0274	Interaction: 0.02911, 1, 85; treatment:151.1, 1, 85; time point: 6.856, 1, 85
	48 h			0.0385	
1*L*	24 h	Control vs 17-AAG	Two-way ANOVA, Sidak'smultiple comparison test	0.9726	Interaction: 0.5439, 1, 86; treatment:80.21, 1, 86; time point: 6.856, 1, 85
	48 h			0.4136	
1-1*B*	Polar metabolites	Control vs 17-AAG	Unpaired *t* test	Listed in ExtendedData Table 1-1	N/A
1-1*D*	Fatty acids		Unpaired *t* test	Listed in ExtendedData Table 1-2	N/A
1-1*F*		Control vs CHX	One-way ANOVA, Dunnett'smultiple comparison test	0.0142	9.046, 2, 6
		Control vs 17-AAG		0.8498	
1-1*H*		Control vs 17-AAG	Unpaired *t* test with Welch'scorrection	0.3747	1.388, 32, 41
2*B*	1 d, Without photobleaching:Control vs 1 d, Without photobleaching:17-AAG		Two-way ANOVA, Turkey'smultiple comparison test	0.0198	Interaction: 6.99, 3, 6; treatment:6.916, 1, 2; time point: 10.88, 3, 6
	1 d, Without photobleaching:Control vs 3 d, Without photobleaching:Control			0.6093	
	1 d, Without photobleaching:Control vs 3 d, Without photobleaching:17-AAG			0.0318	
	1 d, Without photobleaching:Control vs 1 d, Photobleachingof aneural AChR cluster:Control			0.0069	
	1 d, Without photobleachingControl vs 1 d, Photobleachingof aneural AChR cluster:17-AAG			0.004	
	1 d, Without photobleaching:Control vs 3 d, Photobleachingof aneural AChR cluster:Control			0.0011	
	1 d, Without photobleaching:Control vs 3 d, Photobleaching of aneural AChR cluster:17-AAG			0.0018	
	1 d, Without photobleaching:17-AAG vs 3 d, Without photobleaching:Control			0.126	
	1 d, Without photobleaching:17-AAG vs 3 d, Without photobleaching:17-AAG			0.9991	
	1 d, Without photobleaching:17-AAG vs 1 d, Photobleaching of aneural AChR cluster:Control			0.8927	
	1 d, Without photobleaching:17-AAG vs 1 d, Photobleaching of aneural AChR cluster:17-AAG			0.5569	
	1 d, Without photobleaching:17-AAG vs 3 d, Photobleaching of aneural AChR cluster:Control			0.0818	
	1 d, Without photobleaching:17-AAG vs 3 d, Photobleaching of aneural AChR cluster:17-AAG			0.1828	
	3 d, Without photobleaching:Control vs 3 d, Without photobleaching:17-AAG			0.2166	
	3 d, Without photobleaching:Control vs 1 d, Photobleaching of aneuralAChR cluster:Control			0.0356	
	3 d, Without photobleaching:Control vs 1 d, Photobleaching of aneural AChR cluster:17-AAG			0.018	
	3 d, Without photobleaching:Control vs 3 d, Photobleaching of aneural AChR cluster:Control			0.004	
	3 d, Without photobleaching:Control vs 3 d, Photobleaching of aneural AChR cluster:17-AAG			0.007	
	3 d, Without photobleaching:17-AAG vs 1 d, Photobleaching of aneural AChR cluster:Control			0.6672	
	3 d, Without photobleaching:17-AAG vs 1 d, Photobleaching of aneural AChR cluster:17-AAG			0.3446	
	3 d, Without photobleaching:17-AAG vs 3 d, Photobleaching of aneural AChR cluster:Control			0.0487	
	3 d, Without photobleaching:17-AAG vs 3 d, Photobleaching of aneural AChR cluster:17-AAG			0.1064	
	1 d, Photobleaching of aneural AChR cluster:Control vs 1 d, Photobleaching of aneural AChR cluster:17-AAG			0.9916	
	1 d, Photobleaching of aneural AChR cluster:Control vs 3 d, Photobleaching of aneural AChR cluster:Control			0.3032	
	1 d, Photobleaching of aneural AChR cluster:Control vs 3 d, Photobleaching of aneural AChR cluster:17-AAG			0.6183	
	1 d, Photobleaching of aneural AChR cluster:17-AAG vs 3 d, Photobleaching of aneural AChR cluster:Control			0.6054	
	1 d, Photobleaching of aneural AChR cluster:17-AAG vs 3 d, Photobleaching of aneural AChR cluster:17-AAG			0.9315	
	3 d, Photobleaching of aneural AChR cluster:Control vs 3 d, Photobleaching of aneural AChR cluster:17-AAG			0.9898	
2*C*	1 d, Without photobleaching:Control vs 1 d, Without photobleaching:17-AAG		Two-way ANOVA, Turkey'smultiple comparison test	>0.9999	Interaction: 0.293, 3, 6; treatment:0.2994, 1, 2; time point: 11.74, 3, 6
	1 d, Without photobleaching:Control vs 3 d, Without photobleaching:Control			0.406	
	1 d, Without photobleaching:Control vs 3 d, Without photobleaching:17-AAG			0.6936	
	1 d, Without photobleaching:Control vs 1 d, Photobleaching of aneural AChRcluster:Control			>0.9999	
	1 d, Without photobleaching:Control vs 1 d, Photobleaching of aneural AChRcluster:17-AAG			>0.9999	
	1 d, Without photobleaching:Control vs 3 d, Photobleaching of aneural AChRcluster:Control			0.832	
	1 d, Without photobleaching:Control vs 3 d, Photobleaching of aneural AChR cluster:17-AAG			0.9999	
	1 d, Without photobleaching:17-AAG vs 3 d, Without photobleaching:Control			0.4594	
	1 d, Without photobleaching:17-AAG vs 3 d, Without photobleaching:17-AAG			0.7563	
	1 d, Without photobleaching:17-AAG vs 1 d, Photobleaching of aneural AChRcluster:Control			>0.9999	
	1 d, Without photobleaching:17-AAG vs 1 d, Photobleaching of aneural AChR cluster:17-AAG			>0.9999	
	1 d, Without photobleaching:17-AAG vs 3 d, Photobleaching of aneural AChR cluster:Control			0.8826	
	1 d, Without photobleaching:17-AAG vs 3 d, Photobleaching of aneural AChR cluster:17-AAG			>0.9999	
	3 d, Without photobleaching:Control vs 3 d, Without photobleaching:17-AAG			0.9968	
	3 d, Without photobleaching:Control vs 1 d, Photobleaching of aneural AChRcluster:Control			0.4322	
	3 d, Without photobleaching:Control vs 1 d, Photobleaching of aneural AChR cluster:17-AAG			0.4267	
	3 d, Without photobleaching:Control vs 3 d, Photobleaching of aneural AChR cluster:Control			0.9722	
	3 d, Without photobleaching:Control vs 3 d, Photobleaching of aneural AChR cluster:17-AAG			0.5671	
	3 d, Without photobleaching:17-AAG vs 1 d, Photobleaching of aneural AChR cluster:Control			0.7253	
	3 d, Without photobleaching:17-AAG vs 1 d, Photobleaching of aneural AChR cluster:17-AAG			0.7188	
	3 d, Without photobleaching:17-AAG vs 3 d, Photobleaching of aneural AChR cluster:Control			>0.9999	
	3 d, Without photobleaching:17-AAG vs 3 d, Photobleaching of aneural AChR cluster:17-AAG			0.8595	
	1 d, Photobleaching of aneural AChR cluster:Control vs 1 d, Photobleaching of aneural AChR cluster:17-AAG			>0.9999	
	1 d, Photobleaching of aneural AChR cluster:Control vs 3 d, Photobleaching of aneural AChR cluster:Control			0.8583	
	1 d, Photobleaching of aneural AChR cluster:Control vs 3 d, Photobleaching of aneural AChR cluster:17-AAG			>0.9999	
	1 d, Photobleaching of aneural AChR cluster:17-AAG vs 3 d, Photobleaching of aneural AChR cluster:Control			0.853	
	1 d, Photobleaching of aneural AChR cluster:17-AAG vs 3 d, Photobleaching of aneural AChR cluster:17-AAG			>0.9999	
	3 d, Photobleaching of aneural AChR cluster:Control vs 3 d, Photobleaching of aneural AChR cluster:17-AAG			0.9511	
3*B*		WT vs Control MO	One-way ANOVA, Dunnett'smultiple comparison test	0.1383	21.35, 2,
		WT vs Grp94 MO		0.0089	
3*D*	Top	WT vs Control MO	Two-way ANOVA, Turkey'smultiple comparison test	0.7282	Interaction: 4.283, 4, 18; treatment:127.9, 2, 18; Cluster type:80.22, 2, 18
		WT vs Grp94 MO		0.0002	
		Control MO vs Grp94 MO		0.0008	
	Bottom	WT vs Control MO		0.1487	
		WT vs Grp94 MO		0.0013	
		Control MO vs Grp94 MO		<0.0001	
	Total	WT vs Control MO		0.974	
		WT vs Grp94 MO		<0.0001	
		Control MO vs Grp94 MO		<0.0001	
3*E*	Normalized intensity of AChRat cluster region	WT vs Control MO	One-way ANOVA, Dunnett'smultiple comparison test	0.7416	6.557, 2, 9
		WT vs Grp94 MO		0.0141	
	Ratio of perforated area/entireAChR cluster area	WT vs Control MO		0.8166	31.46, 2, 9
		WT vs Grp94 MO		0.0002	
3*G*		GFP-XAC vs GFP-XAC + Control MO	Two-way ANOVA, Sidak'smultiple comparison test	0.6801	Interaction: 6.469, 2, 45; treatment:80.39, 1, 45; time point: 6.469, 2, 45
		GFP-XAC vs GFP-XAC + Grp94 MO		0.0162	
3*I*	Normalized intensity of AChR at bead contacts	GFP-XAC alone vs GFP-XAC + Control MO	One-way ANOVA, Turkey'smultiple comparison test	0.8926	6.716, 2, 9
		GFP-XAC alone vs GFP-XAC + Grp94 MO		0.0201	
		GFP-XAC + Control MO vs GFP-XAC + Grp94 MO		0.0409	
	Ratio of GFP-XAC intensity at bead/non-bead contact region	GFP-XAC alone vs GFP-XAC + Control MO		0.3488	4.59, 2, 9
		GFP-XAC alone vs GFP-XAC + Grp94 MO		0.0345	
		GFP-XAC + Control MO vs GFP-XAC + Grp94 MO		0.3109	
3*H*	0 h	GFP-XAC alone vs Control MO + GFP-XAC	Two-way ANOVA, Sidak'smultiple comparison test	0.9862	Interaction: 2.583, 2, 29; treatment:18.65, 1, 29; time point: 4.2, 2, 29
		GFP-XAC alone vs Grp94 MO + GFP-XAC		0.0038	
	4 h	GFP-XAC alone vs Control MO + GFP-XAC		0.7729	
		GFP-XAC alone vs Grp94 MO + GFP-XAC		0.9996	
	GFP-XAC alone	0 h vs 4 h		0.0012	
	Control MO + GFP-XAC			0.0299	
	Grp94 MO + GFP-XAC			0.8305	
3-1*B*	XAC	WT vs Control MO	One-way ANOVA, Dunnett'smultiple comparison test	0.1955	12.16, 2, 9
		WT vs Grp94 MO		0.0016	
3-1*C*	p34-Arc	WT vs Control MO		0.9667	7.445, 2, 6
		WT vs Grp94 MO		0.0242	
3-1*D*	Vinculin	WT vs Control MO		0.8551	5.924, 2, 6
		WT vs Grp94 MO		0.0331	
4*C*	Perforated region	Control vs 17-AAG	Unpaired *t* test with Welch'scorrection	0.0025	11.1, 11, 5
	AChR-rich region	Control vs 17-AAG		0.049	11.64, 10, 5
4-1*C*	Perforated region	GFP-XAC vs GFP-XAC (S3A)	One-way ANOVA, Turkey'smultiple comparison test	0.1195	16.04, 2, 27
		GFP-XAC vs GFP-XAC (S3E)		0.0015	
		GFP-XAC (S3A) vs GFP-XAC (S3E)		<0.0001	
	AChR-rich region	GFP-XAC vs GFP-XAC (S3A)		0.0025	16.9, 2, 27
		GFP-XAC vs GFP-XAC (S3E)		0.0497	
		GFP-XAC (S3A) vs GFP-XAC (S3E)		<0.0001	
5*B*		WT vs Control MO	One-way ANOVA, Turkey'smultiple comparison test	0.511	44.59, 2, 6
		WT vs Grp94 MO		0.0003	
		Control MO vs Grp94 MO		0.0007	
5*C*		WT vs Control MO	One-way ANOVA, Turkey'smultiple comparison test	0.9022	8.63, 2, 6
		WT vs Grp94 MO		0.0351	
		Control MO vs Grp94 MO		0.0211	
5*F*		WT vs Control MO	Kruskal–Wallis ANOVA testwith Dunn's multiplecomparison test	N/A	N/A
		WT vs Grp94 MO			
		Control MO vs Grp94 MO			
5*G*		WT vs Control MO	Kruskal–Wallis ANOVA testwith Dunn's multiplecomparison test		
		WT vs Grp94 MO			
		Control MO vs Grp94 MO			
5-1*B*		Control vs 17-AAG	One-way ANOVA, Dunnett'smultiple comparison test	0.0009	13.85, 4, 11
		Control vs PU-WS13		0.0074	
		Control vs Control MO		0.8996	
		Control vs Grp94 MO		0.0004	
5-1*C*		Control vs 17-AAG		0.0195	5.745, 4, 11
		Control vs PU-WS13		0.0233	
		Control vs Control MO		0.9971	
		Control vs Grp94 MO		0.0329	
5-2*B*	0.5 h	Control vs 17-AAG	One-way ANOVA, Dunnett'smultiple comparison test	0.0001	9.548, 2, 74
		Control vs PU-WS13		0.0154	
	4 h	Control vs 17-AAG		0.0001	20.28, 2, 73
		Control vs PU-WS13		0.0001	

## Results

### HSP90 expression and activity regulate AChR cluster formation and stability

**Figure 1.**
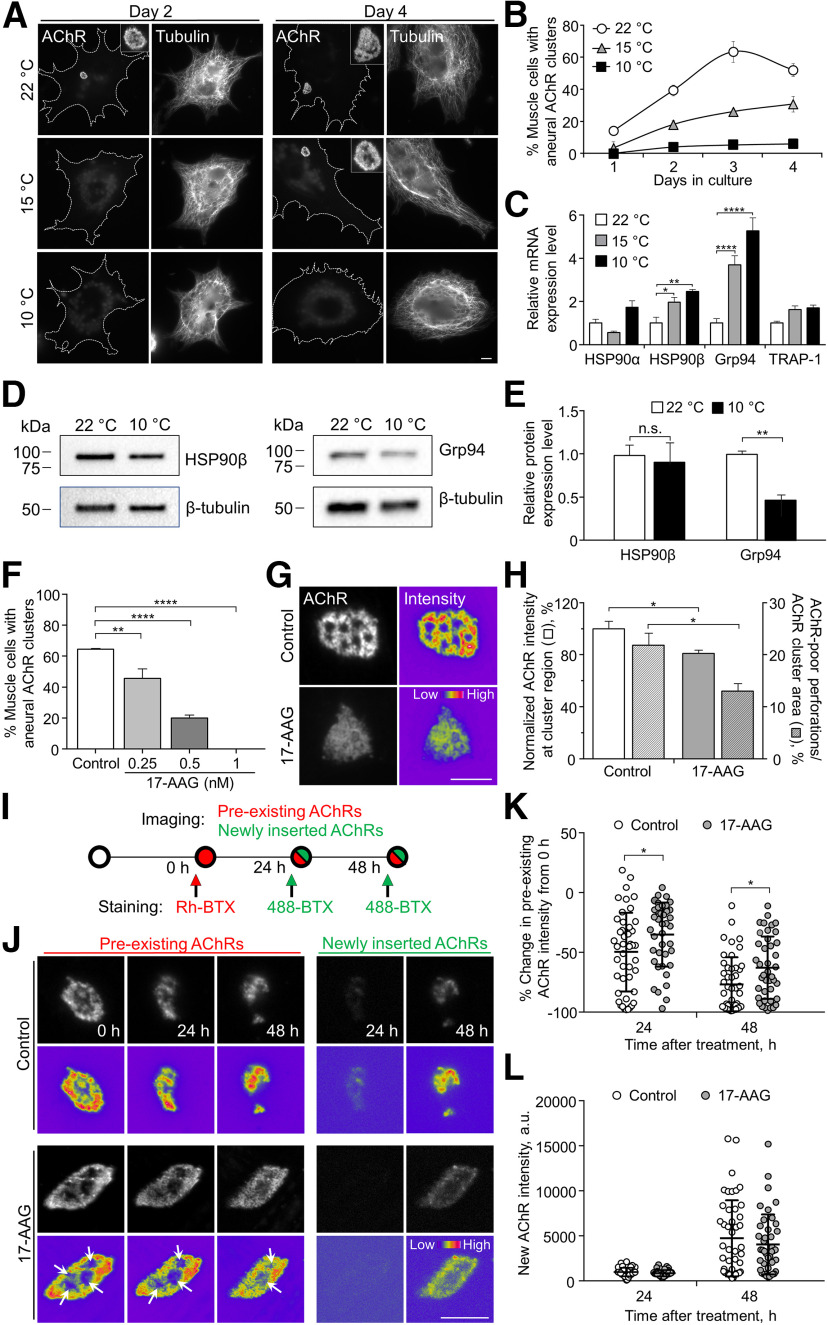
Temperature stress-modulated expression and pharmacological inhibition of HSP90 regulate the formation and stability of aneural AChR clusters. ***A***, Representative images showing the inhibition of aneural AChR cluster formation in cultured *Xenopus* muscle cells treated with lower temperatures. Tubulin immunostaining indicated that cytoskeletal structures were largely unaffected in muscle cells cultured at different temperatures, ranging from 10–22°C. ***B***, Quantification showing the percentage of cultured muscle cells with bottom aneural AChR clusters at different culturing temperatures over 4 d; *n* = 150 cells in each condition from three independent experiments. ***C***, Quantification showing the relative mRNA levels of HSP90α, HSP90β, Grp94, and TRAP-1 in 2-d-old *Xenopus* muscle cells cultured at different temperatures; *n* = 3 independent experiments. ***D***, ***E***, Western blot analysis (***D***) and quantification (***E***) showing the protein expression level of HSP90β and Grp94 in *Xenopus* muscle cells cultured at 22°C or 10°C for 2 d. β-Tubulin was used as the loading control for normalization. ***F***, Quantification showing the dose-dependent effects of 17-AAG on aneural AChR cluster formation in cultured *Xenopus* muscle cells; *n* = 191 (Control), *n* = 198 (0.25 nm 17-AAG), *n* = 199 (0.5 nm 17-AAG), and *n* = 200 (1 nm 17-AAG) muscle cells from four independent experiments. ***G***, Representative images showing the organization and intensity of aneural AChR clusters in response to 17-AAG treatment. 8-bit pseudo-color images highlight the relative fluorescence intensity of AChR clusters in different conditions. ***H***, Quantification showing the effects of 17-AAG on the intensity and complexity of aneural AChR clusters; *n* = 55 (Control) and *n* = 44 (17-AAG) muscle cells from three independent experiments for fluorescence intensity measurement (left *y*-axis); *n* = 76 (Control) and *n* = 48 (17-AAG) muscle cells from four independent experiments for cluster complexity measurement (right *y*-axis). ***I***, Schematic diagram illustrating the differential labeling procedure to identify preexisting (red) and newly inserted (green) AChRs with BTX conjugated with different fluorophores. ***J***, Representative sets of time-lapse images showing the topological changes and fluorescence intensity of pre-existing (left panels) and newly inserted (right panels) AChRs at aneural clusters in control (top panels) or 17-AAG-treated (bottom panels) muscle cells. Arrows indicate the progressive reduction of perforated area in aneural AChR clusters. 8-bit pseudo-color images highlight the change in the fluorescence intensity of the same aneural AChR clusters over 48 h with or without 17-AAG treatment. ***K***, ***L***, Individual value plots showing the percentage change in the fluorescence intensity of pre-existing (***K***) and newly inserted (***L***) AChRs in the same aneural AChR clusters at different time-points between control and 17-AAG-treated cells; *n* = 46 (Control) and *n* = 41 (17-AAG) muscle cells from three independent experiments. Scale bars: 10 μm. Data are shown as mean ± SEM (***B***, ***C***, ***E***, ***F***, ***H***) or mean ± SD (***K***, ***L***). Two-way ANOVA with Tukey’s multiple comparisons test (***B***, ***C***), Student’s *t* test (***E***, ***H***), one-way ANOVA with Tukey’s multiple comparison test (***F***), and two-way ANOVA with Sidak’s multiple comparisons test (***K***, ***L***). *, **, and **** represent *p* ≤ 0.05, 0.01, and 0.0001, respectively. n.s.: non-significant.

As *Xenopus* primary cultures can be maintained in a range of different temperatures, this unique feature allows us to investigate whether temperature stress regulates NMJ development. Here, we incubated dissociated *Xenopus* myotomal tissues plated on coated substratum containing laminin under different culture temperatures (Fig. 1*A*). At the normal culture temperature at 22°C (Peng et al., 1991), we detected an increasing percentage of cultured muscle cells with spontaneously formed aneural AChR clusters over the first 3 d in culture (Fig. 1*B*). Interestingly, the formation of aneural AChR clusters was greatly reduced in muscle cultures incubated at lower temperatures (15°C or 10°C), which showed a temperature-dependent response during the entire 4-d culture period (Fig. 1*A*,*B*). Notably, cultured muscle cells at lower temperatures did not show any obvious changes in the cytoskeletal organization, as evidenced by the integrity of microtubule networks throughout the cells across different temperature groups (Fig. 1*A*). These data suggest that temperature stress causes a specific inhibition on aneural AChR cluster formation, rather than some non-specific cellular structure defects, in *Xenopus* muscle cultures.

The expression of HSP family of proteins could be induced in cells under stressful conditions, including temperature changes (Richter et al., 2010). A previous study showed that the molecular chaperone HSP90β plays a role in AChR cluster formation and maintenance by regulating rapsyn turnover (Luo et al., 2008), suggesting the involvement of stress proteins in regulating NMJ development. To examine if the expression of stress proteins in cultured *Xenopus* muscle cells is affected by low-temperature treatment, we first performed quantitative real-time PCR to examine mRNA levels of several HSP90 family members in muscle cells incubated at different temperatures (Fig. 1*C*). Surprisingly, the expression of HSP90α, the inducible isoform of HSP90, was not significantly changed in low-temperature treatments. Instead, we detected a temperature-dependent increase in the expression of another two HSP90 family members, HSP90β and Grp94, in 10°C muscle cultures, which exhibited 2.5-fold and 5.2-fold increase in HSP90β and Grp94 mRNA levels, respectively, compared with that in 22°C muscle cultures. On the other hand, the expression level of mitochondrial-specific HSP90 protein TRAP-1 remained unchanged in different temperature groups. We then performed Western blot analyses to further investigate whether the protein expressions of HSP90β and Grp94 are affected by low-temperature treatment (Fig. 1*D*,*E*). Surprisingly, in contrast to the increase in HSP90β and Grp94 mRNA levels by temperature stress, the protein level of Grp94, but not HSP90β, was significantly reduced by 53.3 ± 0.06% in cultured muscle cells incubated at 10°C for 2 d (Fig. 1*E*). These results suggested that temperature stress affects the expression of Grp94 that may regulate AChR clustering in cultured muscle cells.

To further investigate the regulation by HSP90 activity in aneural AChR clustering, we treated the cultured muscle cells with 17-AAG, a potent HSP90 inhibitor that alters the conformation of molecular chaperone machinery by inhibiting ATPase activity (Sharp and Workman, 2006). 17-AAG treatment exhibited a dose-dependent inhibition of aneural AChR cluster formation in cultured muscle cells (Fig. 1*F*). In cultured cells treated with 0.25 nm 17-AAG, some aneural AChR clusters were found, but they showed a significant reduction in the fluorescence intensity and topological complexity of perforated aneural AChR clusters (Fig. 1*G*,*H*), suggesting that HSP90 activity is involved in the formation and/or maintenance of topologically complex aneural AChR clusters. Taken together, these findings indicated that HSP90 expression and activity are precisely regulated at the optimal range to facilitate the formation of AChR clusters properly.

Previous studies showed that laminin-induced aneural AChR clusters in C2C12 myotubes can undergo topological transformation, mirroring the progressive morphologic changes in synaptic AChR clusters at NMJs *in vivo* (Kummer et al., 2004; Lee et al., 2009). We next examined whether HSP90 activity also participates in the topological remodeling of aneural AChR clusters. After identifying aneural AChR clusters in 2-d-old muscle cultures, time-lapse imaging was then performed to monitor the dynamic changes in the morphology and intensity of the same AChR clusters in response to 1 nm 17-AAG treatment over 48 h. Specifically, preexisting and newly inserted AChRs were differentially labeled by BTX conjugated with red and green fluorophores, respectively, in accordance with a previously established protocol (Lee et al., 2009) and as illustrated in the schematic diagram (Fig. 1*I*). By examining the preexisting AChRs in control muscles, we observed a gradual dispersal of aneural AChR clusters, together with a reduction in AChR intensity, over a 48 h period (Fig. 1*J*,*K*). However, such spontaneous dispersal of aneural AChR clusters was greatly inhibited in the presence of 17-AAG. In contrast, the intensity of newly inserted AChRs between control and 17-AAG-treated muscle cells showed no significant difference (Fig. 1*J*,*L*). Interestingly, instead of dispersing the entire aneural AChR clusters in control muscle cells, 17-AAG treatment caused a gradual loss of perforations, where podosome-like structures (PLSs) are located (Proszynski et al., 2009), within aneural AChR clusters (Fig. 1*J*, bottom panels, arrows). Considering the involvement of PLSs in AChR endocytosis and topological remodeling of aneural AChR clusters in *Xenopus* primary muscle cultures and C2C12 myotubes (Lee et al., 2009; Proszynski et al., 2009; Yeo et al., 2015), we hypothesized that pharmacological inhibition of HSP90 activity affects the spatial localization of PLSs, leading to the stabilization of aneural AChR clusters. Collectively, our data demonstrated that HSP90 activity is involved in both the formation and topological remodeling/dispersal of aneural AChR clusters in cultured muscle cells.

The HSP90 chaperone machinery is known to be a crucial regulator in maintaining cellular homeostasis under stressful conditions and normal metabolism (Schopf et al., 2017). To rule out the possibility that pharmacological inhibition of HSP90 activity by 17-AAG causes global metabolic changes in cultured muscle cells, we firstly performed GC-MS analysis to compare the levels of various polar metabolites and fatty acids between control and 17-AAG-treated muscle cells. The PCAs showed that these two experimental groups from the same biological sample overlapped in the first principal component of polar metabolites or fatty acids (Extended Data Fig. 1–1*A*,*C***;** Extended Data Table 1-1, Table 1-2). These results supported that 17-AAG treatment does not cause a significant change in the overall metabolite profile of cultured *Xenopus* muscle cells. Second, to eliminate the possible secondary effect of HSP90 inhibition on AChR clustering that is contributed by global changes in protein synthesis, we next used the OPP reagent, followed by click chemistry method to visualize the newly synthesized, nascent peptides/proteins (Liu et al., 2012; Slomnicki et al., 2016; Extended Data Fig. 1-1*E*). We detected a similar level of OPP signals between control and 17-AAG-treated muscle cells (Extended Data Fig. 1-1*F*). In contrast, 25 μm CHX, a well-known protein synthesis inhibitor, showed a significant reduction in OPP signals. Third, to further validate the inhibitory effects on AChR clustering by 17-AAG are not because of defects in surface AChR insertion, we examined the density of single AChR molecules on the muscle surface using quantum dots as previously described (Geng et al., 2009; Extended Data Fig. 1-1*G*). We detected a similar density of surface AChR molecules between control and 17-AAG-treated muscle cells (Extended Data Fig. 1-1*H*), indicating that *de novo* synthesis, followed by surface targeting mechanisms, of AChR proteins are not affected by HSP90 inhibition. Taken together, we provided multiple lines of evidence supporting that HSP90 inhibition causes a specific effect on AChR clustering and remodeling, rather than a global change of different cellular events in cultured muscle cells.

10.1523/ENEURO.0025-20.2020.f1-1 Extended Data Figure 1-1 HSP90 inhibition does not cause non-specific, global changes in cell metabolism and protein expression in cultured muscle cells. ***A***, ***B***, PCA (***A***) and heat map comparison (***B***) showing a panel of different polar metabolites between control and 17-AAG-treated cultured *Xenopus* muscle cells. Control (green circles) and 17-AAG-treated (red circles) samples were not clearly distinguished in the first principal component axis (*x*-axis); *n* = 3 biological samples; *p* values of each polar metabolite examined were shown in Extended Data Table 1-1. ***C***, ***D***, PCA (***C***) and heat map comparison (***D***) showing a panel of different fatty acids between control and 17-AAG-treated cultured *Xenopus* muscle cells. Control (green circles) and 17-AAG-treated (red circles) samples were not clearly distinguished in the first principal component axis (*x*-axis); *n* = 3 biological samples; *p* values of each fatty acid examined were shown in Extended Data Table 1-2. ***E***, Representative images showing no significant change in the amount of nascent peptides/proteins between control and 17-AAG-treated muscle cells, as shown by OPP signals. ***F***, Quantification showing the fluorescence intensity of OPP signals in muscle cells at different experimental groups; *n* = 237 (control), *n* = 245 (CHX), and *n* = 251 (17-AAG) muscle cells from three independent experiments. ***G***, Representative images showing a similar density of quantum dot-labeled single AChR molecules in membrane surface between control and 17-AAG-treated muscle cells. ***H***, Quantification showing the number of single AChR molecules in membrane surface per unit area between control and 17-AAG-treated muscle cells; *n* = 42 (control) and *n* = 33 (17-AAG) muscle cells from three independent experiments. Scale bars: 100 μm (***E***) or 10 μm (***G***). Data are shown as mean ± SEM (***F***) or mean ± SD (***H***). One-way ANOVA with Dunnett’s multiple comparisons test (***F***) and Student’s *t* test (***H***). * represents *p* ≤ 0.05. n.s.: non-significant. Download Figure 1-1, TIF file.

### Both diffuse and aneurally clustered AChRs contribute to the formation of synaptic AChR clusters

**Figure 2.**
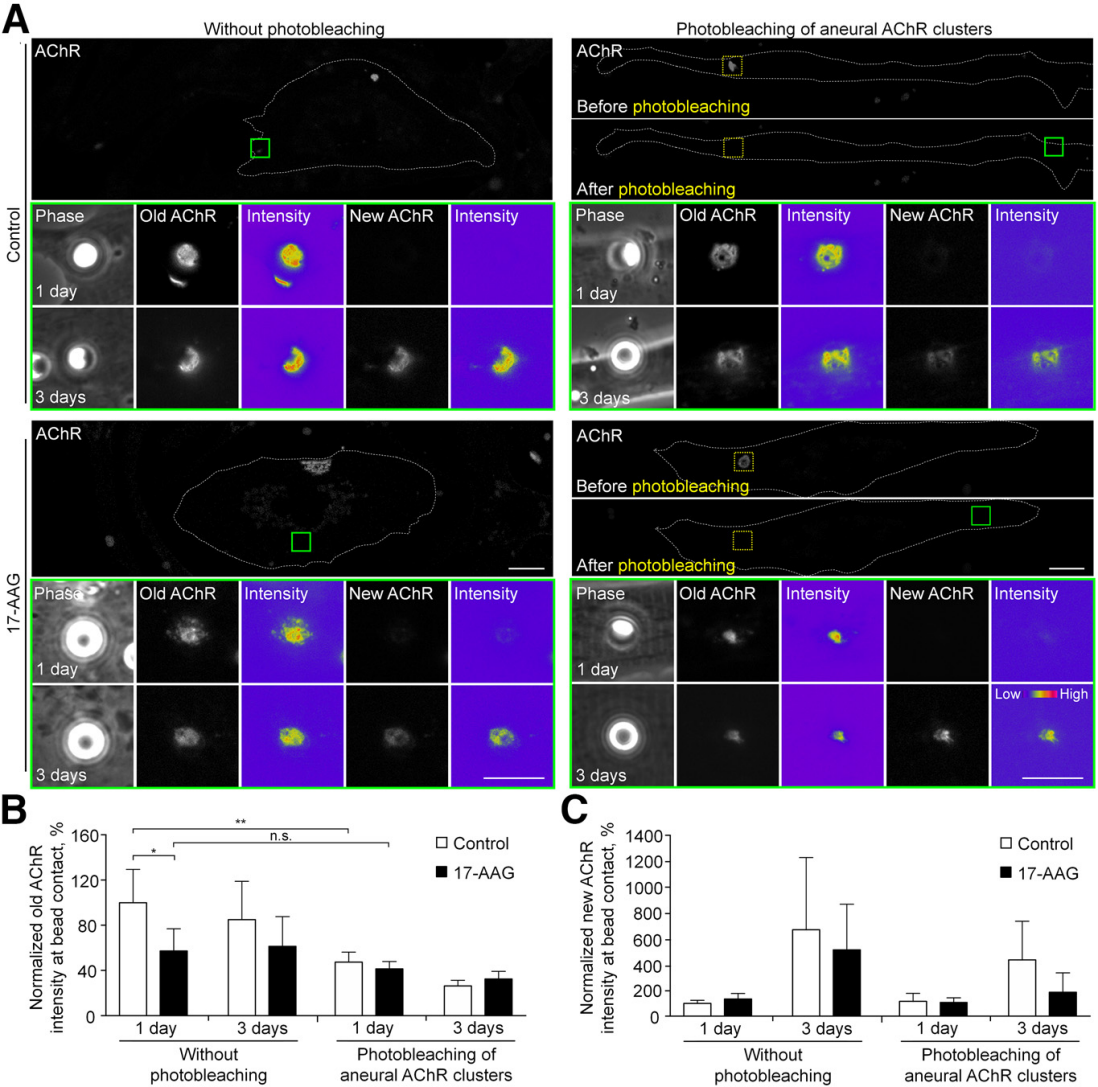
HSP90 regulates AChR recruitment from aneural clusters to agrin-induced clusters. ***A***, Representative images showing the differential contribution of diffuse and aneurally clustered AChRs to agrin bead-induced synaptic AChR clusters in control or 17-AAG-treated muscle cells using laser-based photobleaching approach. Green boxes indicate the magnified view of muscle cells with agrin bead contacts at different time-points for clarity. Yellow dotted-line boxes indicate the photobleaching region of aneural AChR clusters before agrin bead stimulation. Dotted lines highlight the periphery of muscle cells. 8-bit pseudo-color images highlight the relative fluorescence intensity of preexisting (old) and newly inserted (new) AChR signals in muscle cells contacted by agrin beads for 1 and 3 d. ***B***, ***C***, Quantification showing the fluorescence intensity of preexisting (***B***) and newly inserted (***C***) AChR signals at agrin bead-muscle contacts in control or 17-AAG-treated muscle cells, either with or without photobleaching of aneural AChR clusters before agrin bead stimulation; *n* = 11 (control, without photobleaching), *n* = 16 (control, photobleaching of aneural AChR clusters), *n* = 17 (17-AAG-treated, without photobleaching), and *n* = 21 (17-AAG-treated, photobleaching of aneural AChR clusters) muscle cells from three independent experiments. Scale bars: 10 μm. Data are shown as mean ± SEM. Two-way ANOVA with Tukey’s multiple comparisons test. * and ** represent *p* ≤ 0.05 and 0.01, respectively. n.s.: non-significant.

Before synaptogenesis, AChR molecules are present in the surface of muscle fibers (preexisting AChRs) in the forms of aneurally clustered and diffuse receptors. It is believed that nerve innervation of skeletal muscles involves local signals to initiate synaptic AChR cluster formation and global signals to induce aneural AChR cluster dispersal (Dai and Peng, 1998). To demonstrate the differential contribution of aneurally clustered versus diffuse AChR molecules for the assembly of synaptic AChR clusters, laser-based photobleaching experiments were performed (Fig. 2). Specifically, all surface AChRs (diffuse and aneurally clustered ones, referred to as old AChR) were first labeled with Rh-BTX. In the experimental groups, a high-power laser was used to photobleach the fluorescence signals of aneural AChR clusters, while diffuse AChRs in the entire muscle cells were unaffected. Latex beads coated with recombinant agrin were then added onto muscle cells, which has previously been shown to induce AChR clustering at the bead-muscle contacts in a spatially and temporally controllable manner (Lee et al., 2009). After 1- or 3-d agrin bead stimulation, newly synthesized and inserted AChRs (referred to as new AChR) were labeled with 488-BTX. In the experimental group with photobleaching of aneural AChR cluster before agrin bead stimulation, we detected 52.66 ± 0.05% reduction in the intensity of old AChR signals at 1-d agrin bead-muscle contacts, compared with the control group without photobleaching (Fig. 2*A*,*B*). This result suggested that synaptic AChR clusters induced by agrin beads are recruited from both diffuse AChRs and aneural AChR clusters. Furthermore, the contribution of newly inserted AChRs to synaptic AChR clusters was negligible in the first day of agrin bead stimulation (Fig. 2*A*,*C*). Different from previous observations in cultured C2C12 myotubes (Bruneau et al., 2008), our photobleaching approach however did not cause an effect similar to chromophore-assisted light inactivation (CALI) to induce dissipation of the illuminated aneural AChR clusters in cultured *Xenopus* muscle cells labeled with either 594-BTX or Rh-BTX (Extended Data Fig. 2-1). Therefore, our results indicated the differential contribution of aneurally clustered and diffuse AChRs for the assembly of synaptic AChR clusters, rather than the secondary effects caused by photo-dissipation of illuminated AChR clusters and their associated scaffolding proteins.

10.1523/ENEURO.0025-20.2020.f2-1 Extended Data Figure 2-1 No photo-dissipation of illuminated aneural AChR clusters was observed in cultured *Xenopus* muscle cells labeled with Alexa Fluor 594-conjugated BTX. Representative images showing no photo-dissipation effects on illuminated aneural AChR clusters in cultured *Xenopus* muscle cells labeled with either Rh-BTX (left panels) or 594-BTX (right panels). Newly synthesized and inserted AChRs were labeled with 488-BTX at 6 and 24 h after photobleaching. Yellow boxes indicate the photobleaching region covering the entire aneural AChR clusters, while the yellow dotted line box indicates the photobleaching region covering a part of aneural AChR clusters. The recovery of either Rh-BTX or 594-BTX signals was observed at 6 and 24 h after photobleaching the entire (arrows) or partial (arrowheads) region of AChR clusters, respectively. Scale bar: 10 μm. Download Figure 2-1, TIF file.

10.1523/ENEURO.0025-20.2020.f2-2 Extended Data Figure 2-2 HSP90 inhibition stabilizes aneural AChR clusters and their associated rapsyn localization. Representative images showing the stabilization of rapsyn-associated aneural AChR clusters (left panels) and the inhibition of agrin bead-induced synaptic AChR cluster formation (right panels) by 17-AAG treatment. After 4–8 h of agrin bead stimulation, reduced rapsyn signals were detected at dispersing AChR clusters in control muscle cells. In contrast, rapsyn was highly localized at stabilized aneural AChR clusters in 17-AAG-treated muscle cells. At the agrin bead-muscle contacts, agrin-induced AChR clusters were associated with rapsyn localization in control muscle cells but not in 17-AAG-treated muscle cells. Scale bars: 5 μm. Download Figure 2-2, TIF file.

To further examine whether HSP90 regulates the recruitment of both diffuse AChRs and aneural AChR clusters to the nascent postsynaptic sites, the same laser-based photobleaching experiment was performed using muscle cells pretreated with 1 nm 17-AAG for 1 h before the experiment. A similar level of old AChR signals was detected at agrin bead-muscle contacts in 17-AAG-treated muscle cells either with or without photobleaching the aneural AChR clusters, suggesting that 17-AAG treatment abolishes the recruitment of aneural AChR clusters, not diffuse AChRs, to the synaptic sites (Fig. 2*A*,*B*). On the other hand, the signals of new AChRs at agrin bead-muscle contacts showed no significant difference between control and 17-AAG-treated muscle cells (Fig. 2*A*,*C*). As HSP90β plays a role in NMJ by regulating rapsyn localization and turnover (Luo et al., 2008), our immunostaining experiments further showed the association of rapsyn to those aneural AChR clusters that were stabilized by 17-AAG treatment in agrin bead-contacted muscles (Extended Data Fig. 2–2). In contrast, reduced rapsyn localization was detected at dispersing AChR clusters in control muscle cells after 4–8 h agrin bead stimulation. Taken together, these data suggested that aneural AChR clusters and diffuse AChRs contribute equally to the assembly of synaptic AChR clusters, and the recruitment of aneural AChR clusters, but not diffuse or newly inserted AChRs, to agrin-induced synaptic clusters is dependent on HSP90 activity and rapsyn turnover.

### Grp94 regulates ADF/cofilin-associated AChR redistribution on agrin stimulation

**Figure 3.**
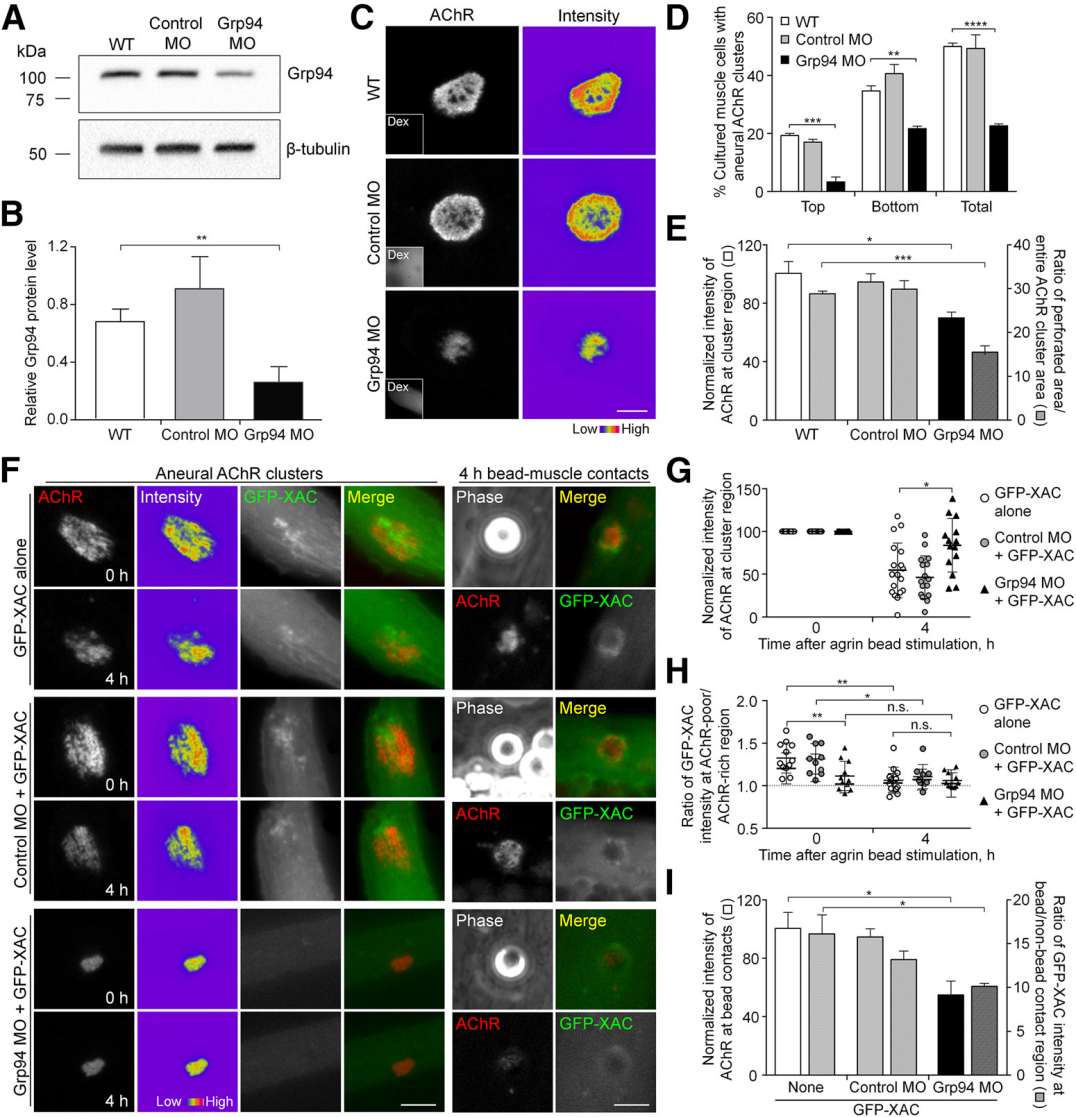
Grp94 knock-down inhibits agrin bead-induced AChR clustering by modulating ADF/cofilin localization. ***A***, ***B***, Western blot analysis (***A***) and quantification (***B***) showing the expression level of Grp94 in WT, Control MO, and Grp94 MO embryos. β-Tubulin was used as the loading control for normalization. ***C***, Representative images showing the intensity and complexity of aneural AChR clusters in response to Grp94 knock-down. 8-bit pseudo-color images highlight the relative fluorescence intensity of AChR clusters in control versus Grp94 knock-down muscle cells. Insets show the fluorescent dextran (Dex) signals, indicating the presence of MO. ***D***, Quantification showing the effects of MO-mediated Grp94 knock-down on the formation of aneural AChR clusters in cultured muscles; *n* = 150 (WT), *n* = 143 (Control MO), and *n* = 150 (Grp94 MO) muscle cells from three independent experiments. ***E***, Quantification showing the effects of MO-mediated Grp94 knock-down on the intensity and complexity of aneural AChR clusters; *n* = 64 (WT), *n* = 47 (Control MO), and *n* = 52 (Grp94 MO) muscle cells from four independent experiments for fluorescence intensity measurement (left *y*-axis); *n* = 44 (WT), *n* = 42 (Control MO), and *n* = 44 (Grp94 MO) muscle cells from four independent experiments for cluster complexity measurement (right *y*-axis). ***F***, Representative sets of time-lapse images showing the effects of Grp94 knock-down on GFP-XAC localization in association with the dispersal of aneural AChR clusters (left panels) and with the formation of agrin bead-induced AChR clusters (right panels). 8-bit pseudo-color images highlight the change in fluorescence intensity of aneural AChR clusters after agrin bead stimulation for 4 h. ***G***, ***H***, Individual value plots showing the percentage change in the fluorescence intensities of AChRs (***G***) and GFP-XAC (***H***) in the same aneural AChR clusters among different conditions after agrin bead stimulation for 4 h; *n* = 12 (GFP-XAC alone), *n* = 9 (Control MO + GFP-XAC), and *n* = 11 (Grp94 MO + GFP-XAC) muscle cells from three independent experiments. ***I***, Quantification showing the effects of Grp94 knock-down on agrin bead-induced AChR clustering (left *y*-axis) and GFP-XAC localization (right *y*-axis); *n* = 24 (GFP-XAC alone), *n* = 24 (Control MO + GFP-XAC), and *n* = 21 (Grp94 MO + GFP-XAC) muscle cells from four independent experiments. Scale bars: 5 μm. Data are shown as mean ± SEM (***B***, ***D***, ***E***, ***I***) or mean ± SD (***G***, ***H***). One-way ANOVA with Dunnett’s multiple comparisons test (***B***, ***E***), two-way ANOVA with Tukey’s multiple comparisons test (***D***), two-way ANOVA with Sidak’s multiple comparison test (***G***, ***H***) and one-way ANOVA with Turkey’s multiple comparison test (***I***). *, **, ***, and **** represent *p* ≤ 0.05, 0.01, 0.001, and 0.0001, respectively. n.s.: non-significant.

To investigate whether Grp94 is the HSP90 isoform that regulates aneural AChR cluster formation, we used antisense MO, which showed an effective knock-down of endogenous Grp94 expression level by Western blot analyses (Fig. 3*A*,*B*). Grp94 knock-down caused a significant inhibition in the formation of aneural AChR clusters, including both bottom and top clusters (Fig. 3*D*). Additionally, the intensity and complexity of aneural bottom AChR clusters were significantly reduced in Grp94 knock-down muscle cells (Fig. 3*C*,*E*), in agreement with the observations in 17-AAG-treated muscles (Fig. 1*G*). Given the known function of HSP90β in agrin-induced AChR cluster formation and maintenance (Luo et al., 2008), we here showed that another HSP90 protein Grp94 is required for the initial formation of aneural AChR clusters.

A previous study demonstrated that ADF/cofilin localization is spatiotemporally correlated with AChR redistribution from aneural to agrin-induced clusters (Lee et al., 2009). We next further studied whether Grp94 is required for ADF/cofilin-mediated AChR redistribution. To test this, we performed dual-channel time-lapse imaging to monitor the change of AChR cluster morphology and intensity in correlation with ADF/cofilin localization at aneural AChR clusters in the same muscle cells before and after agrin bead stimulation (Fig. 3*F-I*). Consistent with previous studies (Lee et al., 2009), we also observed spatially localized GFP-XAC to be associated with agrin-induced AChR clusters at the bead-muscle contacts, which was accompanied with the reduction of localized GFP-XAC signals at the dispersing aneural AChR clusters, in muscle cells with GFP-XAC alone or with Control MO + GFP-XAC. However, in Grp94 MO muscle cells, agrin-induced AChR clusters at the bead-contacted sites were significantly reduced, and the morphology and intensity of aneural AChR clusters remained largely unchanged before and after agrin bead stimulation (Fig. 3*F-I*). Importantly, the spatial localization of GFP-XAC associated with aneural and agrin-induced AChR clusters was largely reduced in Grp94 knock-down muscles (Fig. 3*F*,*H*,*I*). Taken together, our data suggested that Grp94 is required for the formation and dispersal of agrin-induced and aneural AChR clusters, respectively, possibly through modulating the spatial localization of ADF/cofilin.

Since ADF/cofilin is also considered as one of the PLS core markers to be associated with AChR clusters (Chan et al., 2020b), the reduced complexity of aneural AChR clusters in Grp94 knock-down muscle cells may be because of the dysregulation of PLS localization. To determine the requirement of Grp94 for the spatial localization of PLSs at aneural AChR clusters, our immunostaining experiments showed that XAC and p34-Arc, one of the Arp2/3 subunits, were spatially localized at the perforations of aneural AChR clusters in WT or Control MO muscles, but not in Grp94 MO muscles (Extended Data Fig. 3-1*A-C*). Likewise, PLS cortex marker vinculin was found to be localized at the edge of perforations within AChR clusters and at the cell periphery of WT or Control MO muscles, but its spatial localization patterns were significantly reduced in Grp94 MO muscles (Extended Data Fig. 3-1*A*,*D*). These results suggested that MO-mediated knock-down of endogenous Grp94 may also affect proper localization of PLSs at aneural AChR clusters, which in turn affect the stability of aneural AChR clusters and their contribution to the synaptic clusters under synaptogenic stimulation.

10.1523/ENEURO.0025-20.2020.f3-1 Extended Data Figure 3-1 Grp94 knock-down affects PLS localization at aneural AChR clusters. ***A***, Representative images showing the effects of Grp94 knock-down on the spatial localization of PLS core markers (XAC and p34-Arc) and cortex marker (vinculin) at aneural AChR clusters. ***B***, Quantification showing the spatial enrichment of XAC at aneural AChR cluster versus non-AChR regions in the same muscle cells; *n* = 44 (WT), *n* = 43 (Control MO), and *n* = 38 (Grp94 MO) muscle cells from four independent experiments. ***C***, Quantification showing the spatial enrichment of p34-Arc at aneural AChR cluster versus non-AChR regions in the same muscle cells; *n* = 27 (WT), *n* = 29 (Control MO), and *n* = 32 (Grp94 MO) muscle cells from three independent experiments. ***D***, Quantification showing the spatial enrichment of vinculin at aneural AChR cluster versus non-AChR regions in the same muscle cells; *n* = 39 (WT), *n* = 33 (Control MO), and *n* = 33 (Grp94 MO) muscle cells from three independent experiments. Scale bars: 10 μm. Data are shown as mean ± SEM. One-way ANOVA with Dunnett’s multiple comparisons test. * and ** represent *p* ≤ 0.05 and 0.01, respectively. Download Figure 3-1, TIF file.

### Grp94 regulates spatial localization and dynamic turnover of ADF/cofilin at aneural AChR clusters

**Figure 4.**
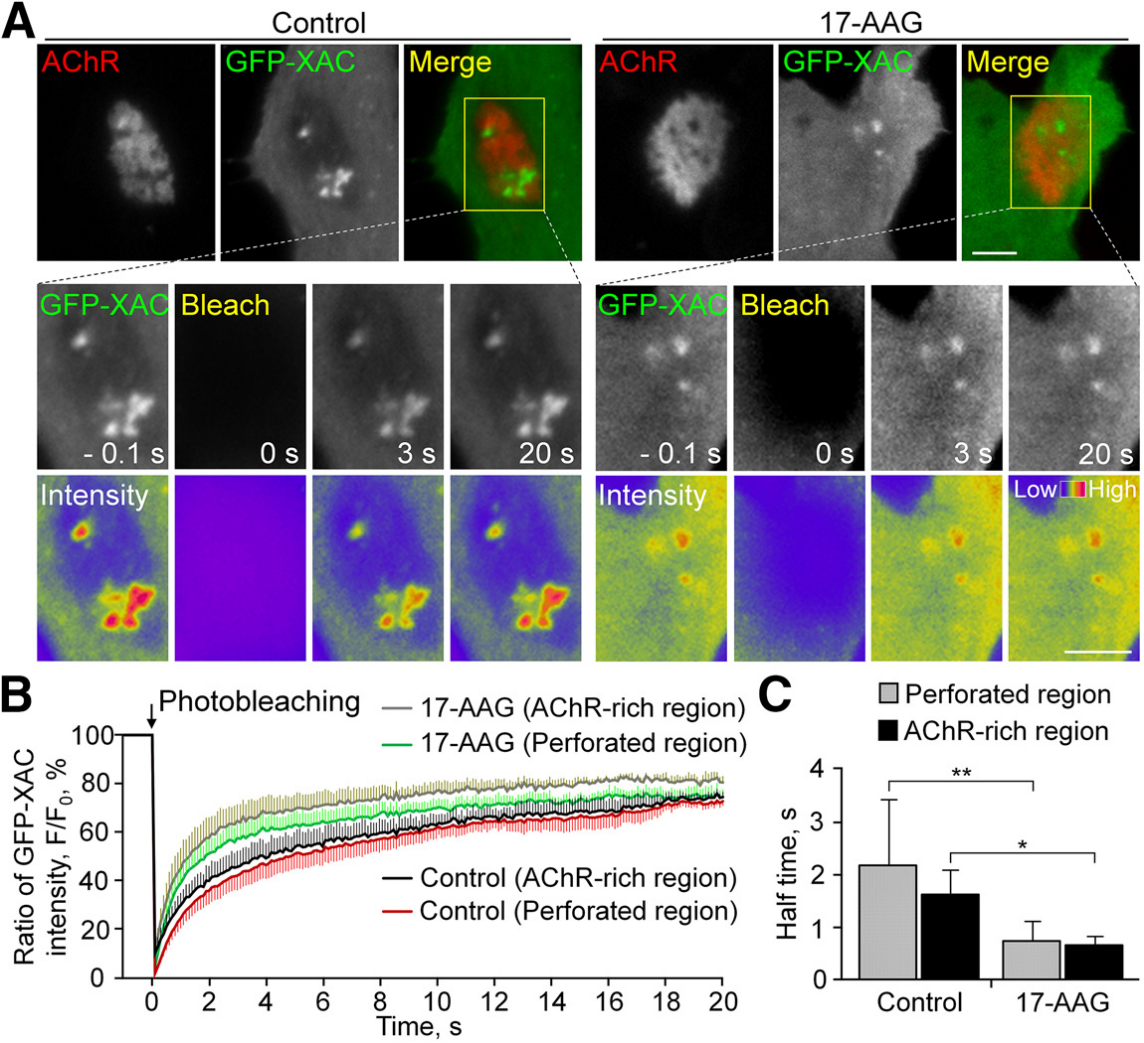
HSP90 inhibition accelerates ADF/cofilin turnover at different regions of aneural AChR clusters. ***A***, Representative time-lapse TIRF images showing the fluorescence recovery of GFP-XAC signals after photobleaching the region of aneural AChR clusters (yellow rectangles, which are magnified in bottom rows with multiple timepoints) in control or 17-AAG-treated muscle cells. 8-bit pseudo-color images highlight the relative fluorescence intensity of GFP-XAC signals. ***B***, ***C***, Quantification showing the FRAP curves (***B***) and the calculated recovery half-time (***C***) of GFP-XAC signals at perforated and AChR-rich regions within aneural AChR clusters in control versus 17-AAG-treated muscle cells; *n* = 12 (Control) and *n* = 7 (17-AAG) muscle cells from three independent experiments. Scale bars: 5 μm. Data are shown as mean ± SEM. Student’s *t* test. * and ** represent *p* ≤ 0.05 and 0.01, respectively.

Since spatially localized ADF/cofilin may direct AChR endocytosis, trafficking, and/or insertion for the assembly of synaptic AChR clusters at developing NMJs via transcytosis (Lee et al., 2009; Yeo et al., 2015), we hypothesized that Grp94 activity regulates the dynamic turnover of localized ADF/cofilin so as to control the stability of aneural AChR clusters. To test this, we performed TIRF imaging and FRAP experiments on GFP-XAC-overexpressing muscle cells either with or without 17-AAG treatment (Fig. 4). Specifically, laser-based photobleaching was conducted at the region of aneural AChR clusters (Fig. 4*A*, yellow rectangles), followed by time-lapse imaging to monitor the recovery of GFP-XAC fluorescence signals in the photobleached region (Fig. 4*A*,*B*). In control muscle cells, we detected a rapid recovery (in seconds after photobleaching) of GFP-XAC signals at aneural AChR clusters. By examining the half-time of fluorescence recovery (the time taken for the fluorescence intensity to recover to half of the plateau level), we found that 17-AAG treatment caused a significant reduction in the recovery half-time of GFP-XAC signals by 59% to 0.86 ± 0.2 s in the perforated regions of AChR clusters (Fig. 4*C*), indicating a much faster turnover rate of ADF/cofilin at aneural AChR clusters on HSP90 inhibition. Interestingly, we also detected a significant reduction in the recovery half-time of GFP-XAC in the AChR-rich region of aneural clusters by 17-AAG treatment, suggesting that HSP90 inhibition increases the turnover of perimembrane fraction of ADF/cofilin at not only the perforated regions, but also the AChR-rich regions, of aneural clusters.

In migrating cells, HSP90 is known to form a molecular complex containing slingshot (SSH), a serine/threonine phosphatase that dephosphorylates and activates ADF/cofilin, to regulate lamellipodial protrusion and directed motility (Fotedar and Margolis, 2015), indicating that HSP90 may modulate the phosphorylation state of ADF/cofilin. To test whether HSP90-regulated ADF/cofilin turnover is mediated through phosphocycling, we performed TIRF-FRAP experiments to compare the fluorescence recovery rate in muscle cells overexpressing different phosphorylation mutant or WT forms of GFP-XAC (Extended Data Fig. 4-1). The constitutively active (S3A) mutant of GFP-XAC exhibited a much slower recovery half-time than the WT form at both perforated and AChR-rich regions of aneural clusters, suggesting that active ADF/cofilin molecules bind to and modulate the dynamic turnover of PLS and cortical actin at perforated and AChR-rich regions of AChR clusters, respectively. Similar to the effects of 17-AAG treatment (Fig. 4*C*), the inactive ADF/cofilin mutant (S3E) showed a significant reduction in the recovery half-time at both perforated and AChR-rich regions of aneural clusters, compared with the WT form. These data suggested that HSP90 may modulate ADF/cofilin activity via phosphorylation at its serine-3 residue.

10.1523/ENEURO.0025-20.2020.f4-1 Extended Data Figure 4-1 ADF/cofilin phosphorylation mutants exhibit differential turnover rates at aneural AChR clusters. ***A***, ***B***, Quantification showing the FRAP curves of GFP-XAC signals at perforated (***A***) and AChR-rich (***B***) regions within aneural AChR clusters in cultured muscle cells over-expressing WT or serine-3 phosphorylation mutant forms (S3A and S3E) of GFP-XAC; *n* = 13 (GFP-XAC), *n* = 8 [GFP-XAC(S3A)], and *n* = 12 [GFP-XAC(S3E)] muscle cells from three independent experiments. **(C)** Quantification showing the calculated recovery half time of GFP-XAC signals at perforated and AChR-rich regions within aneural AChR clusters in cultured muscle cells over-expressing WT or serine-3 phosphorylation mutant forms of GFP-XAC; *n* = 13 (GFP-XAC), *n* = 8 [GFP-XAC(S3A)], and *n* = 12 [GFP-XAC(S3E)] muscle cells from three independent experiments. Data are shown as mean ± SEM. One-way ANOVA with Tukey’s multiple comparisons test. * and ** represent *p* ≤ 0.05 and 0.01, respectively. n.s.: non-significant. Download Figure 4-1, TIF file.

### Postsynaptic Grp94 regulates synaptic structure and function of developing NMJs

**Figure 5.**
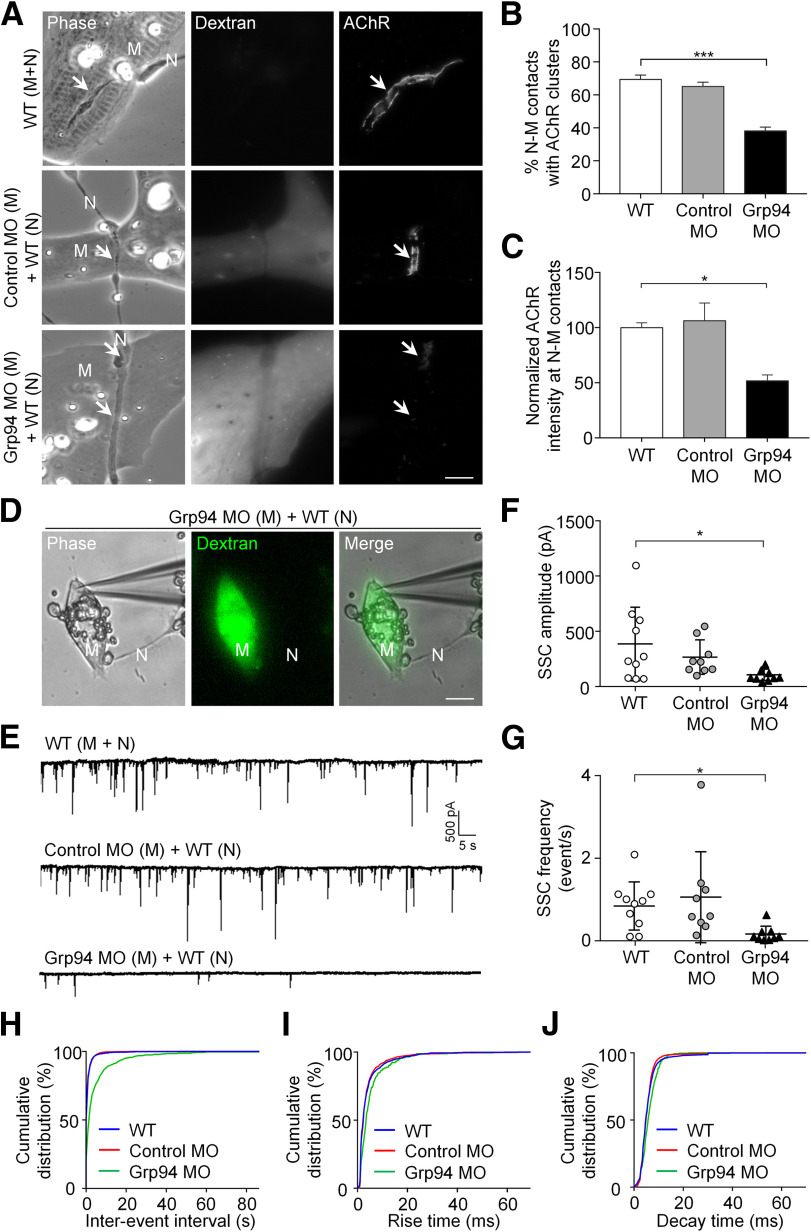
Postsynaptic Grp94 knock-down impairs synaptic structures and functions at developing NMJs. ***A***, Representative images showing the effects of muscle Grp94 knock-down on nerve-induced AChR clustering. Fluorescent dextran signals indicate the presence of MO. Arrows indicate sites of nerve-muscle contacts. ***B***, ***C***, Quantification showing the effects of muscle Grp94 knock-down on the percentage of nerve-muscle contacts with AChR clusters (***B***) and the fluorescence intensity of nerve-induced AChR clusters (***C***) in 1-d-old *Xenopus* nerve-muscle co-cultures; *n* = 150 (WT), *n* = 146 (Control MO), and *n* = 191 (Grp94 MO) nerve-muscle contacts from three independent experiments for quantifying the percentage of nerve-muscle contacts with synaptic AChR clusters (***B***); *n* = 33 (WT), *n* = 31 (Control MO), and *n* = 33 (Grp94 MO) nerve-muscle contacts from three independent experiments for measuring AChR fluorescence intensity (***C***). ***D***, Representative images showing the whole-cell patch-clamp recording on a Grp94 MO muscle cell innervated by a WT spinal neuron. Fluorescent dextran signals indicate the presence of MO. ***E***, Representative electrophysiological recording traces of SSCs recorded from WT, Control MO, or Grp94 MO muscles that were innervated by WT spinal neurons. ***F***, ***G***, Quantification showing the effects of muscle Grp94 knock-down on the amplitude (***F***) and frequency (***G***) of SSCs. ***H–J***, Cumulative distribution plots of the inter-event interval (***H***), 10–90% rise time (***I***), and decay time (***J***) of SSCs recorded from WT, Control MO, or Grp94 MO muscles innervated by WT spinal neurons. *n* = 10 (WT), *n* = 9 (Control MO), and *n* = 9 (Grp94 MO) nerve-muscle pairs from three independent experiments (***F–J***). Scale bars: 10 μm. “M”: muscle; “N”: neuron. Data are represented as mean ± SEM (***B***, ***C***) and mean ± SD (***F***, ***G***). One-way ANOVA with Tukey’s multiple comparisons test (***B***, ***C***). Kruskal–Wallis ANOVA test with Dunn's multiple comparison test (***F***, ***G***). * and *** represent *p* ≤ 0.05 and 0.001, respectively.

To examine the roles of postsynaptic Grp94 in the formation of NMJs *in vitro*, we examined nerve-induced AChR clustering in *Xenopus* nerve-muscle co-cultures by knocking down the endogenous expression of muscle Grp94 specifically. In WT nerve-muscle co-cultures [WT (M + N)] or in the chimeric co-cultures containing Control MO muscles and WT neurons [Control MO (M) + WT (N)], nerve-induced synaptic AChR clusters were highly concentrated along the nerve-muscle contact sites (Fig. 5*A*). In contrast, we observed a significant reduction in the percentage of nerve-muscle contacts with AChR clusters and the fluorescence intensity of nerve-induced AChR clusters in the chimeric co-cultures containing Grp94 MO muscles and WT neurons [Grp94 MO (M) + WT (N)] (Fig. 5*A-C*). These data suggested that Grp94 is required for the formation of nerve-induced AChR clusters, consistent with the requirement of Grp94 for agrin bead-induced AChR clustering as shown above (Fig. 3*F*). To determine whether pharmacological inhibition of HSP90 or molecular manipulation of Grp94 expression affects the spatial localization of rapsyn that leads to the reduced density of synaptic AChR clusters, we performed rapsyn immunostaining in nerve-muscle co-cultures treated with different HSP90 inhibitors or in different chimeric co-cultures (Extended Data Fig. 5-1). In WT [WT (M + N)] or in the chimeric co-cultures containing Control MO muscles and WT neurons [Control MO (M) + WT (N)], rapsyn was spatially colocalized with nerve-induced synaptic AChR clusters (Extended Data Fig. 5-1*A*). However, significant reductions in AChR intensity and its associated rapsyn signals were detected in co-cultures treated with 17-AAG or PU-WS13, a specific Grp94 inhibitor. Similar observations were made in the chimeric co-cultures containing Grp94 MO muscles and WT neurons [Grp94 MO (M) + WT (N)] (Extended Data Fig. 5-1), indicating muscle Grp94 regulates the assembly of nerve-induced synaptic AChR clusters by modulating the precise localization of postsynaptic scaffold protein rapsyn.

10.1523/ENEURO.0025-20.2020.f5-1 Extended Data Figure 5-1 HSP90 inhibition or Grp94 knock-down suppresses nerve-induced synaptic AChR clusters with reduced rapsyn localization. ***A***, Representative images showing the effects of HSP90 inhibition or muscle Grp94 knock-down on nerve-induced AChR clustering and rapsyn localization at nerve-muscle contact sites. Dotted lines indicate nerve-muscle contacts. Insets show fluorescent dextran signals as cell-lineage tracer. “M”: muscle; “N”: neuron. ***B***, ***C***, Quantifications showing the fluorescence intensity of synaptic AChR clusters (***B***) and rapsyn (***C***) along the nerve-muscle contacts in 1-d-old *Xenopus* nerve-muscle co-cultures in the presence or absence of 17-AAG or PU-WS13 and in the chimeric co-cultures of WT neurons and muscle cells with Control MO or Grp94 MO; *n* = 26 (Control), *n* = 9 (17-AAG), *n* = 10 (PU-WS13), *n* = 18 (Control MO), and *n* = 14 (Grp94 MO) from four independent experiments. Scale bar: 10 μm. Data are shown as mean ± SEM. One-way ANOVA with Dunnett’s multiple comparisons test. *, **, and *** represent *p* ≤ 0.05, 0.01, and 0.001, respectively. n.s.: non-significant. Download Figure 5-1, TIF file.

To further understand the role of HSP90 or Grp94 in AChR redistribution from aneural to synaptic AChR clusters, we next examined the amount of internalized AChR vesicles at aneural AChR clusters on agrin bead stimulation in the presence of 17-AAG or PU-WS13 (Extended Data Fig. 5-2*A*). Compared with control cells, HSP90 or Grp94 inhibition significantly reduced the number of internalized AChR vesicles at aneural AChR clusters in agrin bead-contacted muscle cells (Extended Data Figs. 5-2*A*,*B*). Taken together, our findings suggested that Grp94 is the specific HSP90 family member that regulates the redistribution and recruitment of aneural AChR clusters to the postsynaptic specializations, likely through ADF/cofilin-mediated transcytosis mechanism as previously proposed (Lee et al., 2009).

10.1523/ENEURO.0025-20.2020.f5-2 Extended Data Figure 5-2 Grp94 inhibition reduces the amount of AChR vesicles at aneural clusters in agrin-stimulated muscle cells. ***A***, Representative images showing the effects of 17-AAG or PU-WS13 on AChR internalization at aneural clusters upon agrin stimulation. Images of aneural AChR clusters were taken from a single focal plane (surface AChR), while the maximal projection of intracellular AChR signals was constructed of a stack of 11 images at 0.2 μm per frame (internal AChR). ***B***, Quantification showing the effects of 17-AAG or PU-WS13 on AChR internalization at aneural clusters upon agrin stimulation for 0.5 or 4 h; *n* = 27 (Control, 0.5 h), *n* = 28 (17-AAG, 0.5 h), *n* = 23 (PU-WS13, 0.5 h), *n* = 19 (Control, 4 h), *n* = 28 (17-AAG, 4 h), and *n* = 29 (PU-WS13, 4 h) muscle cells from three independent experiments. Scale bar: 5 μm. Data are shown as mean ± SD. One-way ANOVA with Dunnett’s multiple comparisons test. * and **** represent *p* ≤ 0.05 and 0.0001, respectively. Download Figure 5-2, TIF file.

10.1523/ENEURO.0025-20.2020.t1-1 Extended Data Table 1-1 A list of *p* values in comparing the relative amount of polar metabolites between control and 17-AAG-treated muscle cells. Download Table 1-1, DOCX file.

10.1523/ENEURO.0025-20.2020.t1-2 Extended Data Table 1-2 A list of *p* values in comparing the relative amount of fatty acids between control and 17-AAG-treated muscle cells. Download Table 1-2, DOCX file.

To determine the effects of postsynaptic Grp94 knock-down on synaptic functions of developing NMJs, we performed whole-cell voltage-clamp recordings to examine the SSCs in 1-d-old *Xenopus* nerve-muscle co-cultures (Fig. 5*D*). In the chimeric co-cultures of Grp94 MO (M) + WT (N), we detected a significant reduction in the amplitude of SSCs (Fig. 5*E*,*F*), which is likely attributed by the reduced AChR density at the nerve-muscle contacts as observed in our cell imaging studies (Fig. 5*A*). On the other hand, we also detected a significant reduction in SSC frequency, leading to a right shift pattern in the cumulative distribution of inter-event intervals (Fig. 5*G*,*H*). In contrast, postsynaptic Grp94 knock-down caused no significant effects on the rise time and decay time of SSCs (Fig. 5*I*,*J*), demonstrating that the channel properties of AChRs in muscles remain unaffected. In summary, this study identified postsynaptic Grp94 as a novel regulator to control the synaptic structures and functions at developing NMJs.

## Discussion

Temperature has long been considered as a physiological factor that affects neuronal growth and maintains synaptic homeostasis by modulating presynaptic and postsynaptic elements at the invertebrate NMJs (Tsai et al., 2012; Yeates et al., 2017; Zhu et al., 2018). South African clawed toad, *Xenopus laevis*, is an ectotherm vertebrate that has been widely used as an excellent animal model for embryology studies (Hobson, 1965). Since the body temperature of *Xenopus* is subjected to fluctuations in environmental temperature, this provides an ideal model for studying the molecular mechanisms underlying temperature-dependent alterations in the structure and functions of the vertebrate NMJs. In this study, we provided evidence showing that temperature stress-modulated Grp94 expression and activity regulate the recruitment of aneural AChR clusters for the assembly of postsynaptic specializations through modulating ADF/cofilin activity, suggesting a novel role of Grp94 in the formation of vertebrate NMJs. Consistent with a previous study showing the function of cytosolic HSP90 member, HSP90β, for regulating rapsyn turnover and agrin-induced AChR cluster formation (Luo et al., 2008), our present study further identified another HSP90 family member Grp94, an ER-resident molecular chaperone, in regulating AChR clustering and remodeling during NMJ development.

HSP90 proteins are implicated in diverse biological processes, in which a variety of coordinated regulatory mechanisms are involved to control their expression and activity (Prodromou, 2016). In response to stressful conditions, heat shock factor 1 is an important regulator responsible for the transcriptional regulation of HSP90 genes. In this study, mRNA levels of HSP90β and Grp94 were upregulated in cultured *Xenopus* muscle cells by low-temperature treatment (Fig. 1*C*). However, Grp94 protein level was found to be significantly reduced under low-temperature stress (Fig. 1*D*,*E*). This discrepancy between mRNA and protein levels of Grp94 could be explained by a negative feedback mechanism (DiDomenico et al., 1982; Bader et al., 2015), in which Grp94 mRNA may be upregulated to compensate a reduced amount of Grp94 protein level. As both temperature stress and HSP90 inhibitor significantly suppress the formation of aneural AChR clusters, this suggests that HSP90 expression and activity are essential for the regulation of AChR clustering in cultured muscle cells.

One common concern with all HSP90 studies is that pharmacological inhibition of HSP90 activity may be causing non-specific, global changes in cell metabolism and protein expression. Here, we employed multiple experimental approaches to show that it is unlikely the case in the present study: targeted metabolomics (polar metabolites and fatty acids) study (Extended Data Figs. 1-1*A-D*), nascent protein synthesis assay (Extended Data Fig. 1-1*E*,*F*), single AChR molecule labeling (Extended Data Fig. 1-1*G*,*H*), and newly inserted AChR labeling (Fig. 2*C*). Together, results from all these experiments indicated that HSP90 inhibition by 17-AAG causes a specific effect on AChR clustering and remodeling, rather than a plethora of changes in different cellular events.

Previous genetic studies demonstrated that nerve-independent formation of AChR prepatterns can be detected in the central region of muscle fibers at developing NMJs *in vivo* (Yang et al., 2000, 2001; Lin et al., 2001). Similarly, spontaneously formed aneural AChR clusters can be found in cultured muscle cells, suggesting muscle-intrinsic mechanisms underlying initial AChR cluster formation. Upon synaptogenic induction, it is hypothesized that the dispersal of aneural AChR clusters is temporally coupled with the formation of synaptic AChR clusters (Dai and Peng, 1998). A previous study performed by single AChR tracking approach showed the contribution of surface AChR molecules, derived from either aneural clusters or diffuse receptor pool, to the sites of nerve-muscle contacts (Geng et al., 2009). Interestingly, a recent study further provided a definite evidence to demonstrate the recruitment of aneural AChR clusters for the assembly of nerve-induced synaptic AChR clusters (Chan et al., 2020b). Consistent with that, our present study confirmed the differential contribution of preexisting AChRs (from aneural clusters and diffuse AChRs) versus newly synthesized and inserted AChRs for the assembly of agrin-induced postsynaptic specializations (Fig. 2), in which HSP90 or Grp94 is required for the recruitment of AChRs from aneural clusters, but not from diffuse nor newly inserted ones. The stability of AChR clusters is known to be regulated by rapsyn, a multidomain synaptic adaptor protein for AChR anchoring by interacting directly with actin or indirectly with various cytoskeletal regulatory proteins (Wang et al., 1999; Oury et al., 2019; Xing et al., 2020). Apart from serving as a scaffolding molecule at NMJs, rapsyn contains E3 ligase activity that increases neddylation of AChR subunits (Li et al., 2016), leading to the stabilization of AChR clusters. Consistent with this notion, we observed the association of rapsyn with aneural AChR clusters that are stabilized by HSP90 inhibition (Extended Data Fig. 2-2), preventing AChR molecules of aneural clusters from being recruited to the synaptic sites.

It is worth noting that our laser-based photobleaching experiments did not cause photo-dissipation of illuminated AChR clusters and their intracellular scaffolding proteins (Extended Data Fig. 2-1), as previously observed in cultured C2C12 myotubes (Bruneau et al., 2008). Although the exact molecular mechanism underlying photo-dissipation of AChR clusters remains unknown, illumination of a photosensitizer chromophore (e.g., Alexa Fluor 594) may generate reactive oxygen species, leading to limited damage of surrounding target proteins through CALI. In early *Xenopus* embryos, there are various genetically regulated enzymes involved in antioxidant defences (Rizzo et al., 2007). These enzymes may dampen the possible effects of photo-dissipation in cultured *Xenopus* muscle cells. Therefore, our experimental approach allows us to study the essential roles of AChR molecules derived from dispersing aneural AChR clusters, rather than the destruction and removal of illuminated AChR clusters and their associated scaffolding proteins, in the assembly of agrin-induced synaptic AChR clusters.

**Figure 6.**
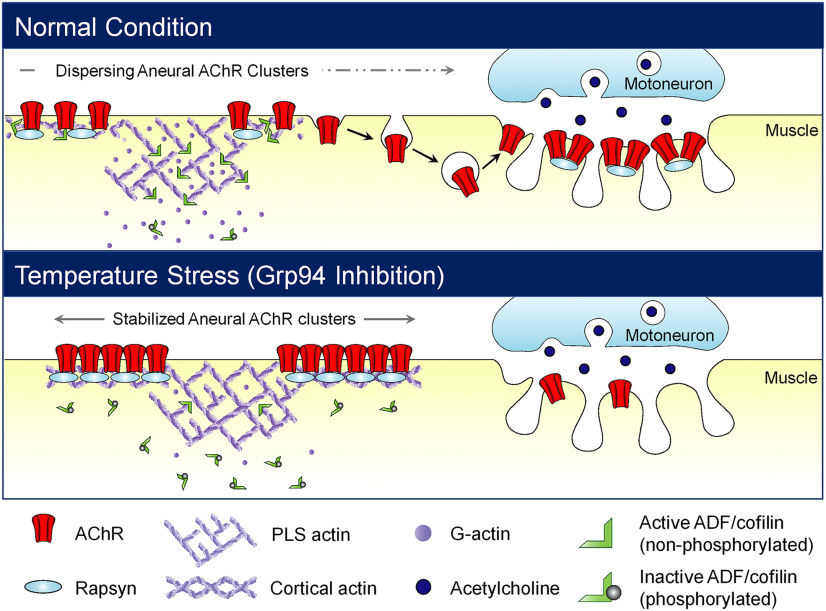
Temperature stress-induced Grp94 inhibition affects AChR recruitment from aneural to synaptic clusters by modulating ADF/cofilin phosphorylation and activity. To allow AChR redistribution during neuromuscular synaptogenesis, modulation of actin dynamics at the cell cortex and at the PLS are required for mobilizing AChR molecules and facilitating vesicular trafficking of AChR molecules at aneural clusters, respectively. Our findings suggest that temperature stress-induced Grp94 inhibition promotes phosphorylation or suppress dephosphorylation of ADF/cofilin at perforated and AChR-rich regions of aneural clusters, thereby stabilizing them against agrin-induced dispersal and recruitment to the postsynaptic sites.

PLS is composed of a core domain containing F-actin and its associated proteins such as ADF/cofilin, Arp2/3 complex, and cortactin, as well as a cortex domain containing focal adhesion proteins such as talin, vinculin, and paxillin. Apart from actin-binding proteins, a recent study also showed that a microtubule-binding protein, microtubule-actin cross linking factor 1 (Macf1), is concentrated at PLS within AChR clusters (Oury et al., 2019). Among different PLS proteins, ADF/cofilin is known to regulate AChR endocytosis, trafficking, and/or insertion at developing NMJs (Lee et al., 2009; Yeo et al., 2015). The actin-binding activity of ADF/cofilin is tightly controlled by a balancing act of phosphorylation and dephosphorylation on its serine-3 residue by LIM or testicular (TES) kinases and SSH phosphatase, respectively. Active, non-phosphorylated ADF/cofilin mediates actin depolymerization by binding to and severing F-actin, while serine-3 phosphorylated ADF/cofilin inhibits its binding to G-actin and F-actin (Bernstein and Bamburg, 2010). As a previous study indicated, constitutively active (S3A) mutant of GFP-XAC is highly localized at the perforated regions of aneural AChR clusters compared with inactive (S3E) mutant or WT forms of GFP-XAC (Lee et al., 2009), suggesting that active ADF/cofilin molecules are preferentially localized at perforated regions of AChR clusters to spatially modulate actin dynamics. Surprisingly, we found that 17-AAG treatment caused a significant increase in the turnover rate of GFP-XAC at both perforated and AChR-rich regions of aneural clusters (Fig. 4), suggesting that ADF/cofilin modulates actin dynamics at not only the PLS, but also the cell cortex in association with AChR molecules. Cortical actin filaments are organized as a dense meshwork that lies directly underneath the plasma membrane (Chugh and Paluch, 2018), where ADF/cofilin-mediated dynamics of cortical actin filaments may facilitate endocytosis and exocytosis events in vesicular trafficking of AChR molecules (Lee et al., 2009, 2014). Similar to the effects of HSP90 inhibition, we observed an increased turnover rate in muscle cells expressing phospho-mimic inactive S3E mutant of GFP-XAC (Extended Data Fig. 4-1), suggesting that HSP90 inhibition may promote phosphorylation or suppress dephosphorylation of ADF/cofilin at perforated and AChR-rich regions of aneural clusters. The phosphorylated ADF/cofilin is inactive in actin binding, therefore it is incapable of modulating the dynamics of both cortical actin (for mobilizing AChR molecules) and PLS actin (for directing vesicular trafficking of AChR molecules) at aneural clusters (Fig. 6).

As HSP90 is known to form a molecular complex containing SSH in migrating cells (Fotedar and Margolis, 2015), HSP90 may indirectly modulate the phosphorylation state and turnover rate of ADF/cofilin in cultured muscle cells. Apart from the phosphorylation regulation, a recent study has identified that cofilin is a physiologically relevant neddylation target that modulates cytoskeletal actin dynamics in neuronal outgrowth (Vogl et al., 2020). Since rapsyn contains E3 ligase activity (Li et al., 2016), it would be of interest to determine whether rapsyn regulates the dynamic turnover of ADF/cofilin via neddylation at AChR clusters. In addition, the spatial localization of ADF/cofilin at the postsynaptic sites can also be regulated by 14-3-3ζ (Lee et al., 2009). As ER stress inducers are known to modulate the expression levels of 14-3-3ζ and Grp94 in hippocampal neurons (Murphy et al., 2008; Brennan et al., 2013), whether temperature stress-induced alterations in Grp94 expression and activity affect 14-3-3ζ-regulated ADF/cofilin localization at AChR clusters remain to be examined. Taken together, we speculate that Grp94 inhibition or knock-down may affect either the dynamic turnover (via phosphocycling and/or neddylation) or the spatial localization (via 14-3-3ζ) of ADF/cofilin, which in turn controls actin-mediated vesicular trafficking of AChRs in the formation of NMJs. Future studies will be focused on elucidating the detailed molecular mechanisms underlying how Grp94 regulates the upstream regulators of ADF/cofilin activity and localization in aneural versus synaptic AChR clusters at developing NMJs.

In the electrophysiological recordings, we showed a significant reduction of SSC frequency in the chimeric co-cultures of Grp94 knock-down muscles and WT neurons (Fig. 5*G*). This effect may be attributed by the reduced probability of neurotransmitter release, suggesting that postsynaptic Grp94 may also be involved in retrograde signaling to affect presynaptic functions. As Grp94 is one of the major Ca^2+^ binding proteins at the ER (Van et al., 1989), it has to contend during the fluctuations in free Ca^2+^ in the lumen, as protein-bound Ca^2+^ is released through the ER membrane channels to the cytosol in response to the physiological demands of the cell (Marzec et al., 2012). Previous studies identified that the activity of Ca^2+^/calmodulin-dependent protein kinase II (CaMKII) in postsynaptic muscles retrogradely modulates the neurotransmitter release at *Drosophila* NMJs (Haghighi et al., 2003). Additionally, conditional deletion of Grp94 results in the loss of β-catenin signaling in the intestinal epithelium (Liu et al., 2013). While muscle β-catenin is known to retrogradely regulate presynaptic differentiation and function at NMJs (Li et al., 2008), it will be of interest to investigate whether postsynaptic Grp94 serves as a regulator of CaMKII-mediated or β-catenin-mediated retrograde signaling to affect presynaptic structure and function.

In summary, this study provides the first evidence suggesting that temperature stress regulates the development of vertebrate NMJs through the expression and activity of postsynaptic Grp94. It is important to note that auto-antibodies against Grp94 has recently been identified in myasthenia gravis (MG), an autoimmune NMJ disease (Suzuki et al., 2011). Another recent study has also shown a significant positive correlation between the age of MG onset and the expression level of Grp78, another ER chaperone (Iwasa et al., 2014). Therefore, results of our study provide insights into not only the fundamental mechanisms underlying the vertebrate NMJ development, but also the pathogenic mechanisms underlying ER stress response and NMJ disruption in MG.

## Synthesis

Reviewing Editor: Niraj Desai, NINDS Decisions are customarily a result of the Reviewing Editor and the peer reviewers coming together and discussing their recommendations until a consensus is reached. When revisions are invited, a fact-based synthesis statement explaining their decision and outlining what is needed to prepare a revision will be listed below. The following reviewer(s) agreed to reveal their identity: Mohammed Akaaboune, Ruth Herbst.

The reviewers and I agree that the revisions address the previous comments well and that the manuscript advances the field. But please address these additional comments that a new reviewer had:

(1) Grp94 mRNA is increased but Grp94 protein is decreased. The authors argue that there is a negative feedback mechanism. Is that known? If yes, please reference or provide other examples where such a feedback loop exists.

(2) Figure 4 is a bit confusing. The authors state that GFP-XAC has a reduced recovery in AChR-rich and perforated regions in the presence of 17-AAG. GFP-XAC expression appears highly upregulated in cells treated with 17-AAG (4B). Why is that? How are the calculations done when there is an almost uniform GFP-XAC signal?

(3) The text requires some editing, such as: predispositions / articles: e.g. line 50, 51, 64, 421, 480, 716 typos: 553 Western blot: capital letter since a proper name

(4) GFP-XAC is not explained in the introduction.

(5) Discussion is too long. Please shorten e.g. 624-635 or 659-677

## Author Response

Author’s Responses (#eN-CFN-0025-20R1)

Point-to-point responses to reviewers’ comments:

1. Grp94 mRNA is increased but Grp94 protein is decreased. The authors argue that there is a negative feedback mechanism. Is that known? If yes, please reference or provide other examples where such a feedback loop exists. We thank the reviewer for raising this question. The negative feedback regulation between mRNA and protein levels has been well documented in yeast (Bader et al., 2015). Interestingly, a previous study has also demonstrated that functional HSP70 proteins are able to repress their own synthesis at both transcriptional and post-transcriptional levels (DiDomenico et al., 1982). Therefore, we hypothesized that Grp94 mRNA may be upregulated in order to compensate a reduced amount of Grp94 protein level. We have now included these two references and have toned down our argument in the discussion section, lines 615-621.

2. Figure 4 is a bit confusing. The authors state that GFP-XAC has a reduced recovery in AChR-rich and perforated regions in the presence of 17-AAG. GFP-XAC expression appears highly upregulated in cells treated with 17-AAG (4B). Why is that? How are the calculations done when there is an almost uniform GFP-XAC signal? We would like to clarify that the expression level of GFP-XAC in muscle cells is largely determined by the amount of GFP-XAC plasmids derived from the microinjected blastomeres in early Xenopus embryos, but is not affected by 17-AAG treatment. In our FRAP experiments, we have measured the half-time of fluorescence recovery (i.e. the time taken for the fluorescence intensity to recover to half of the plateau level) at perforated versus AChR-rich regions of AChR clusters. Our data indicated that 17-AAG treatment caused a significant reduction in the recovery half-time of GFP-XAC signals, suggesting that HSP90 inhibition increases the turnover of perimembrane fraction of ADF/cofilin at not only the perforated regions, but also the AChRrich regions of aneural clusters. We have now further clarified this point in lines 523-528. In the cases when GFP-XAC signals are evenly distributed in the entire AChR clusters, the measured fluorescence signals between perforated and AChR-rich regions are then expected to be at comparable levels.

3. The text requires some editing, such as: predispositions / articles: e.g. line 50, 51, 64, 421, 480, 716; typos: 553; Western blot: capital letter since a proper name We apologize for these mistakes, which have been corrected in the revised manuscript.

4. GFP-XAC is not explained in the introduction. We have now spelled out this abbreviation upon first mention in the text, lines 83-84.

5. Discussion is too long. Please shorten e.g. 624-635 or 659-677 We have now trimmed down the respective sections in Discussion as suggested.

2

References Bader DM, Wilkening S, Lin G, Tekkedil MM, Dietrich K, Steinmetz LM, Gagneur J (2015) Negative feedback buffers effects of regulatory variants. Mol Syst Biol 11:785. DiDomenico BJ, Bugaisky GE, Lindquist S (1982) The heat shock response is self-regulated at both the transcriptional and posttranscriptional levels. Cell 31:593-603
