# Supplementary figures and images for: Grp94 Regulates the Recruitment of Aneural AChR Clusters for the Assembly of Postsynaptic Specializations by Modulating ADF/Cofilin Activity and Turnover
Source: eNeuro. 2020 Sep 2;7(5):ENEURO.0025-20.2020. doi: 10.1523/ENEURO.0025-20.2020 (PMC7540925; doi:10.1523/ENEURO.0025-20.2020)

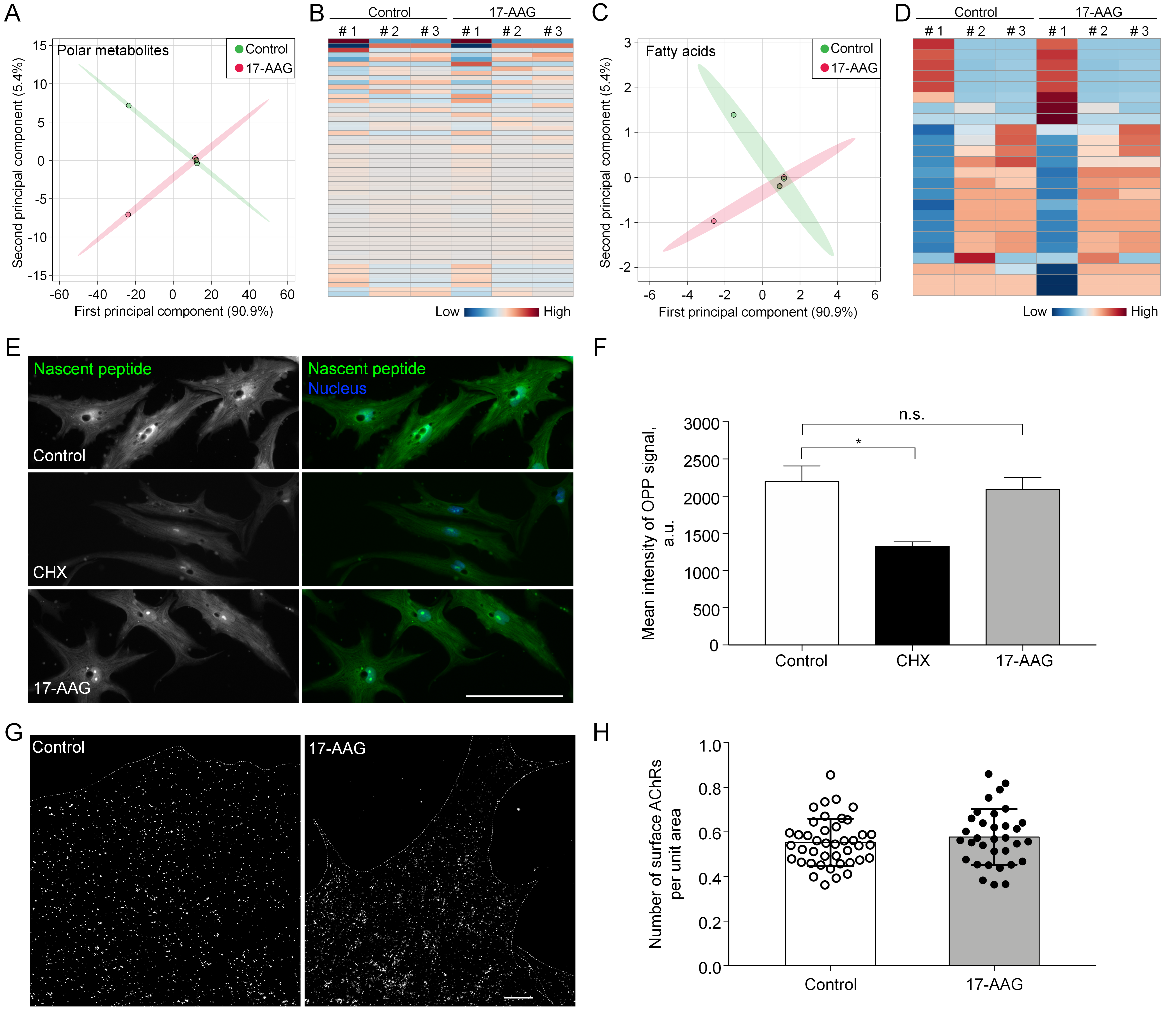

Supplement: Extended Data Figure 1-1 — HSP90 inhibition does not cause non-specific, global changes in cell metabolism and protein expression in cultured muscle cells. A, B, PCA (A) and heat map comparison (B) showing a panel of different polar metabolites between control and 17-AAG-treated cultured Xenopus muscle cells. Control (green circles) and 17-AAG-treated (red circles) samples were not clearly distinguished in the first principal component axis (x-axis); n = 3 biological samples; p values of each polar metabolite examined were shown in Extended Data Table 1-1. C, D, PCA (C) and heat map comparison (D) showing a panel of different fatty acids between control and 17-AAG-treated cultured Xenopus muscle cells. Control (green circles) and 17-AAG-treated (red circles) samples were not clearly distinguished in the first principal component axis (x-axis); n = 3 biological samples; p values of each fatty acid examined were shown in Extended Data Table 1-2. E, Representative images showing no significant change in the amount of nascent peptides/proteins between control and 17-AAG-treated muscle cells, as shown by OPP signals. F, Quantification showing the fluorescence intensity of OPP signals in muscle cells at different experimental groups; n = 237 (control), n = 245 (CHX), and n = 251 (17-AAG) muscle cells from three independent experiments. G, Representative images showing a similar density of quantum dot-labeled single AChR molecules in membrane surface between control and 17-AAG-treated muscle cells. H, Quantification showing the number of single AChR molecules in membrane surface per unit area between control and 17-AAG-treated muscle cells; n = 42 (control) and n = 33 (17-AAG) muscle cells from three independent experiments. Scale bars: 100 μm (E) or 10 μm (G). Data are shown as mean ± SEM (F) or mean ± SD (H). One-way ANOVA with Dunnett’s multiple comparisons test (F) and Student’s t test (H). * represents p ≤ 0.05. n.s.: non-significant. Download Figure 1-1, TIF file. [file enu-eN-CFN-0025-20-s02.tif]

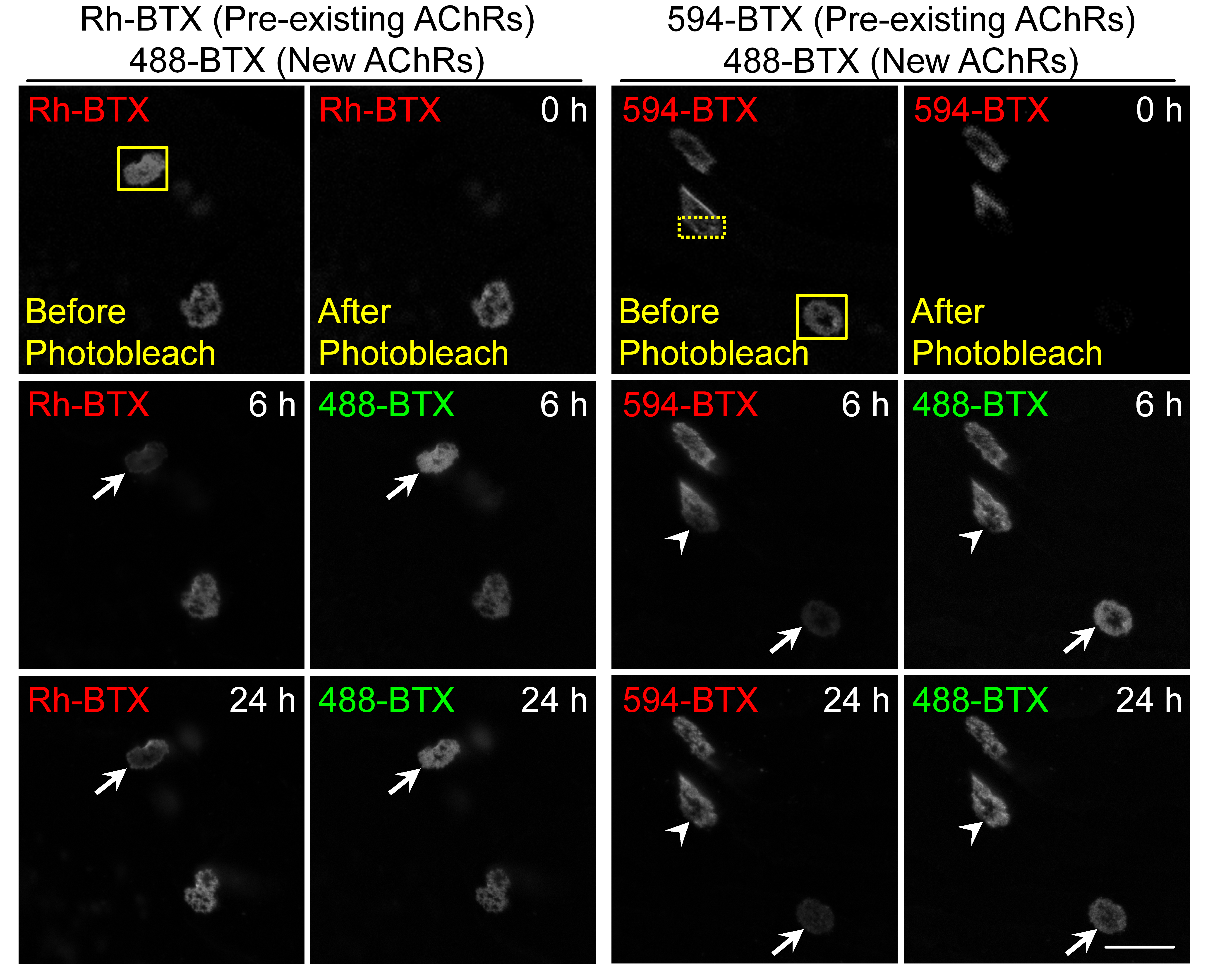

Supplement: Extended Data Figure 2-1 — No photo-dissipation of illuminated aneural AChR clusters was observed in cultured Xenopus muscle cells labeled with Alexa Fluor 594-conjugated BTX. Representative images showing no photo-dissipation effects on illuminated aneural AChR clusters in cultured Xenopus muscle cells labeled with either Rh-BTX (left panels) or 594-BTX (right panels). Newly synthesized and inserted AChRs were labeled with 488-BTX at 6 and 24 h after photobleaching. Yellow boxes indicate the photobleaching region covering the entire aneural AChR clusters, while the yellow dotted line box indicates the photobleaching region covering a part of aneural AChR clusters. The recovery of either Rh-BTX or 594-BTX signals was observed at 6 and 24 h after photobleaching the entire (arrows) or partial (arrowheads) region of AChR clusters, respectively. Scale bar: 10 μm. Download Figure 2-1, TIF file. [file enu-eN-CFN-0025-20-s05.tif]

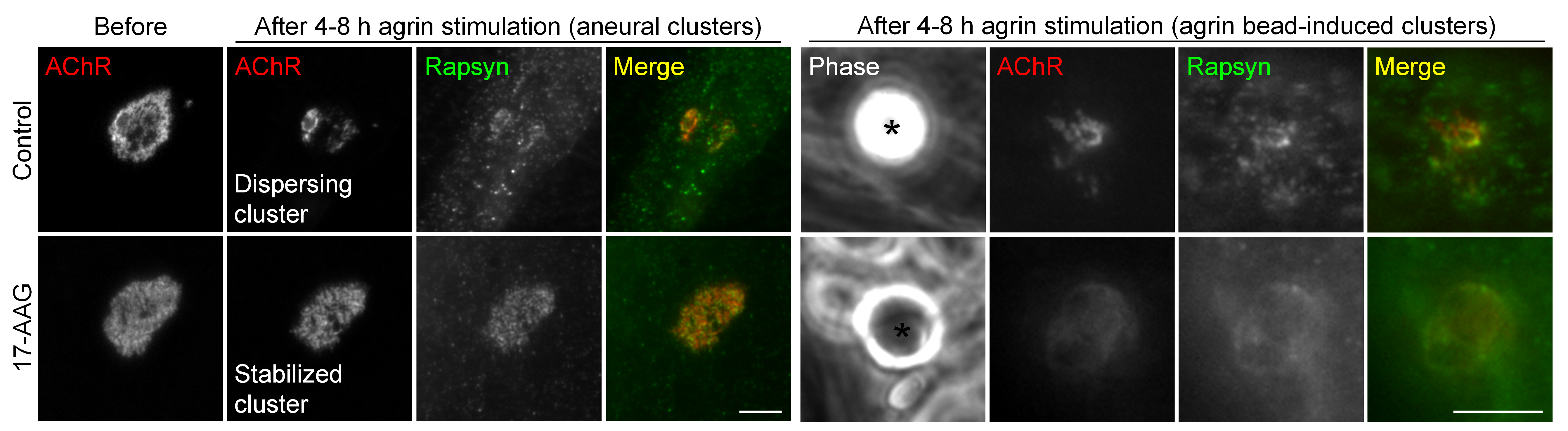

Supplement: Extended Data Figure 2-2 — HSP90 inhibition stabilizes aneural AChR clusters and their associated rapsyn localization. Representative images showing the stabilization of rapsyn-associated aneural AChR clusters (left panels) and the inhibition of agrin bead-induced synaptic AChR cluster formation (right panels) by 17-AAG treatment. After 4–8 h of agrin bead stimulation, reduced rapsyn signals were detected at dispersing AChR clusters in control muscle cells. In contrast, rapsyn was highly localized at stabilized aneural AChR clusters in 17-AAG-treated muscle cells. At the agrin bead-muscle contacts, agrin-induced AChR clusters were associated with rapsyn localization in control muscle cells but not in 17-AAG-treated muscle cells. Scale bars: 5 μm. Download Figure 2-2, TIF file. [file enu-eN-CFN-0025-20-s06.tif]

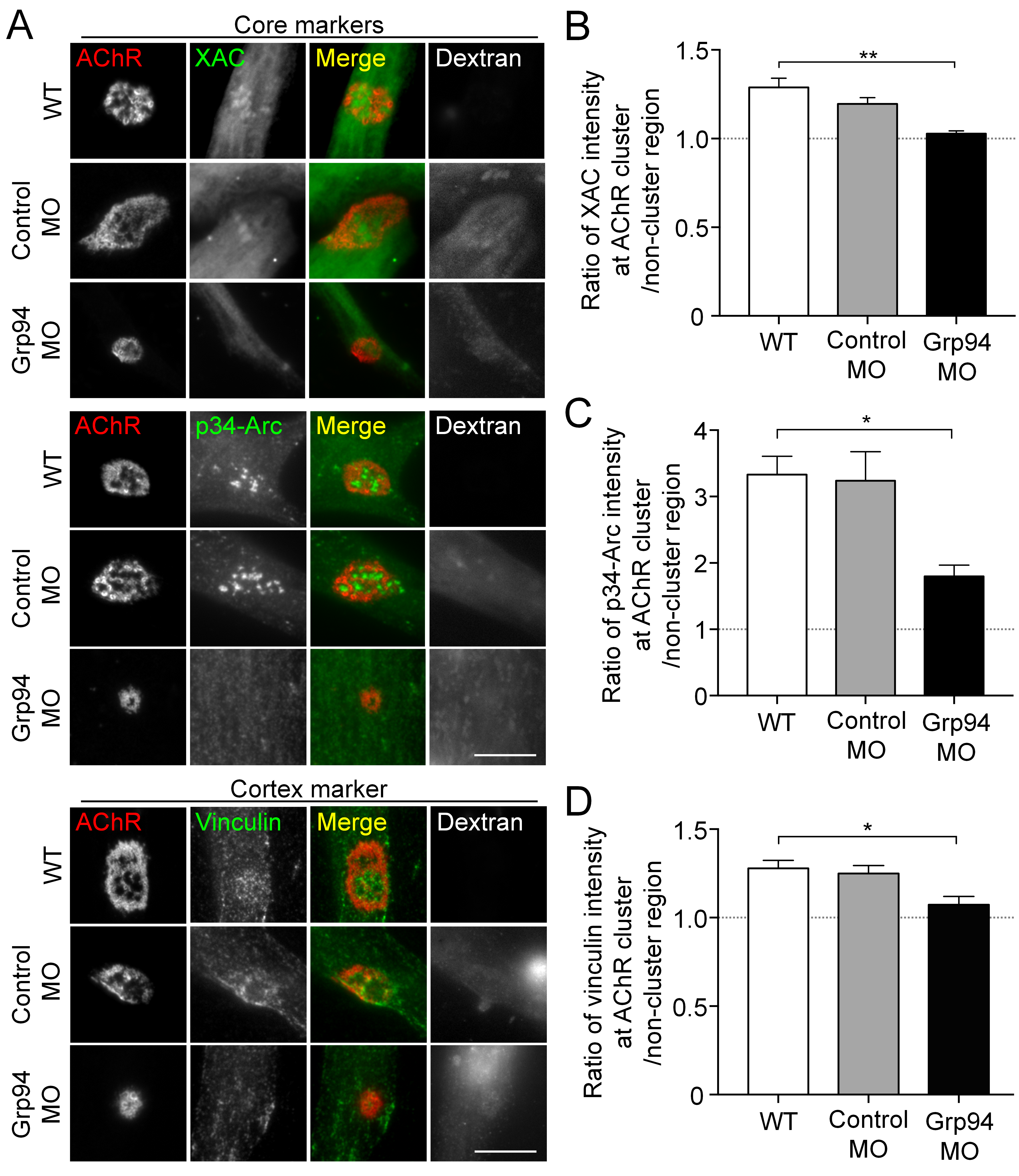

Supplement: Extended Data Figure 3-1 — Grp94 knock-down affects PLS localization at aneural AChR clusters. A, Representative images showing the effects of Grp94 knock-down on the spatial localization of PLS core markers (XAC and p34-Arc) and cortex marker (vinculin) at aneural AChR clusters. B, Quantification showing the spatial enrichment of XAC at aneural AChR cluster versus non-AChR regions in the same muscle cells; n = 44 (WT), n = 43 (Control MO), and n = 38 (Grp94 MO) muscle cells from four independent experiments. C, Quantification showing the spatial enrichment of p34-Arc at aneural AChR cluster versus non-AChR regions in the same muscle cells; n = 27 (WT), n = 29 (Control MO), and n = 32 (Grp94 MO) muscle cells from three independent experiments. D, Quantification showing the spatial enrichment of vinculin at aneural AChR cluster versus non-AChR regions in the same muscle cells; n = 39 (WT), n = 33 (Control MO), and n = 33 (Grp94 MO) muscle cells from three independent experiments. Scale bars: 10 μm. Data are shown as mean ± SEM. One-way ANOVA with Dunnett’s multiple comparisons test. * and ** represent p ≤ 0.05 and 0.01, respectively. Download Figure 3-1, TIF file. [file enu-eN-CFN-0025-20-s07.tif]

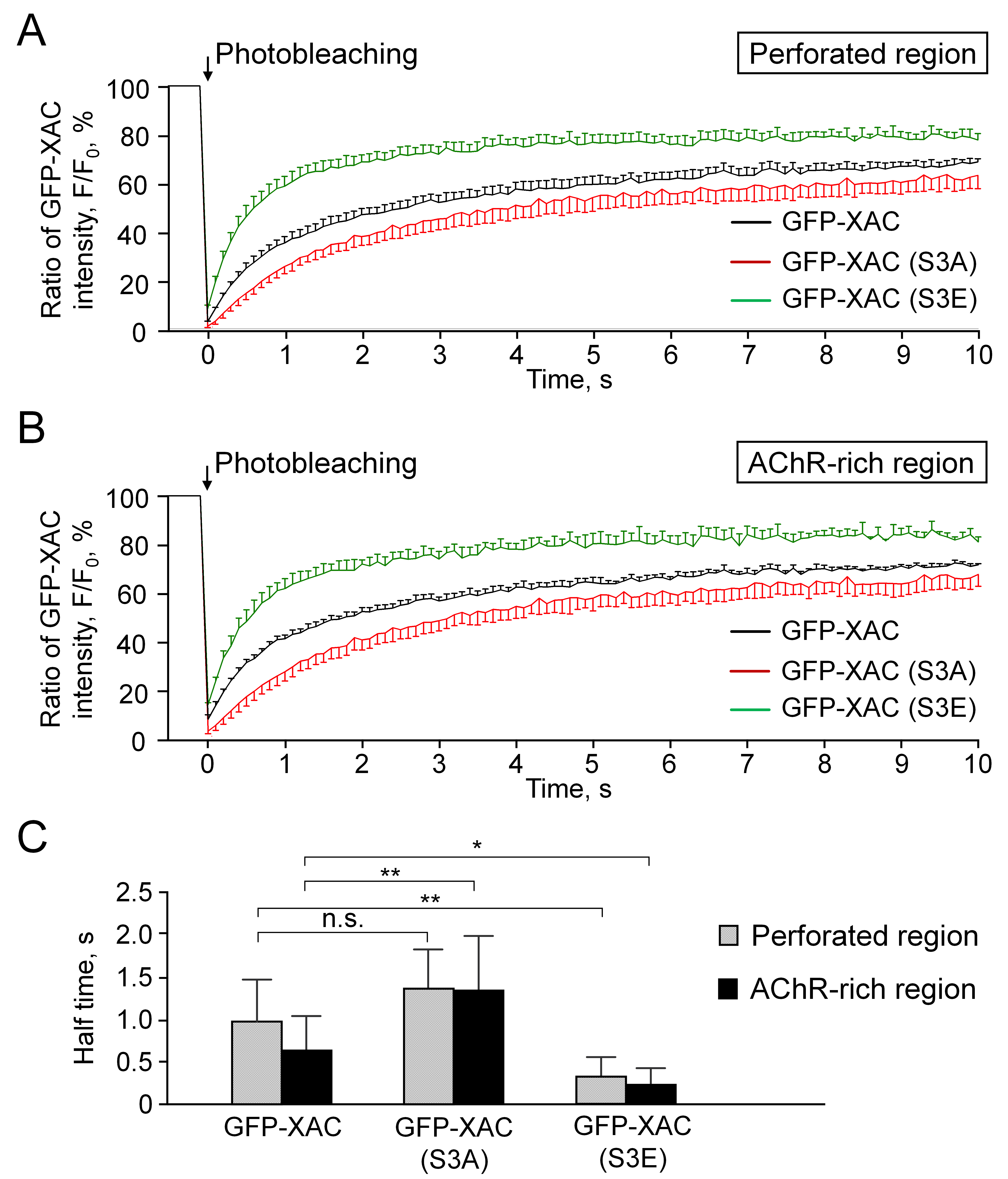

Supplement: Extended Data Figure 4-1 — ADF/cofilin phosphorylation mutants exhibit differential turnover rates at aneural AChR clusters. A, B, Quantification showing the FRAP curves of GFP-XAC signals at perforated (A) and AChR-rich (B) regions within aneural AChR clusters in cultured muscle cells over-expressing WT or serine-3 phosphorylation mutant forms (S3A and S3E) of GFP-XAC; n = 13 (GFP-XAC), n = 8 [GFP-XAC(S3A)], and n = 12 [GFP-XAC(S3E)] muscle cells from three independent experiments. (C) Quantification showing the calculated recovery half time of GFP-XAC signals at perforated and AChR-rich regions within aneural AChR clusters in cultured muscle cells over-expressing WT or serine-3 phosphorylation mutant forms of GFP-XAC; n = 13 (GFP-XAC), n = 8 [GFP-XAC(S3A)], and n = 12 [GFP-XAC(S3E)] muscle cells from three independent experiments. Data are shown as mean ± SEM. One-way ANOVA with Tukey’s multiple comparisons test. * and ** represent p ≤ 0.05 and 0.01, respectively. n.s.: non-significant. Download Figure 4-1, TIF file. [file enu-eN-CFN-0025-20-s08.tif]

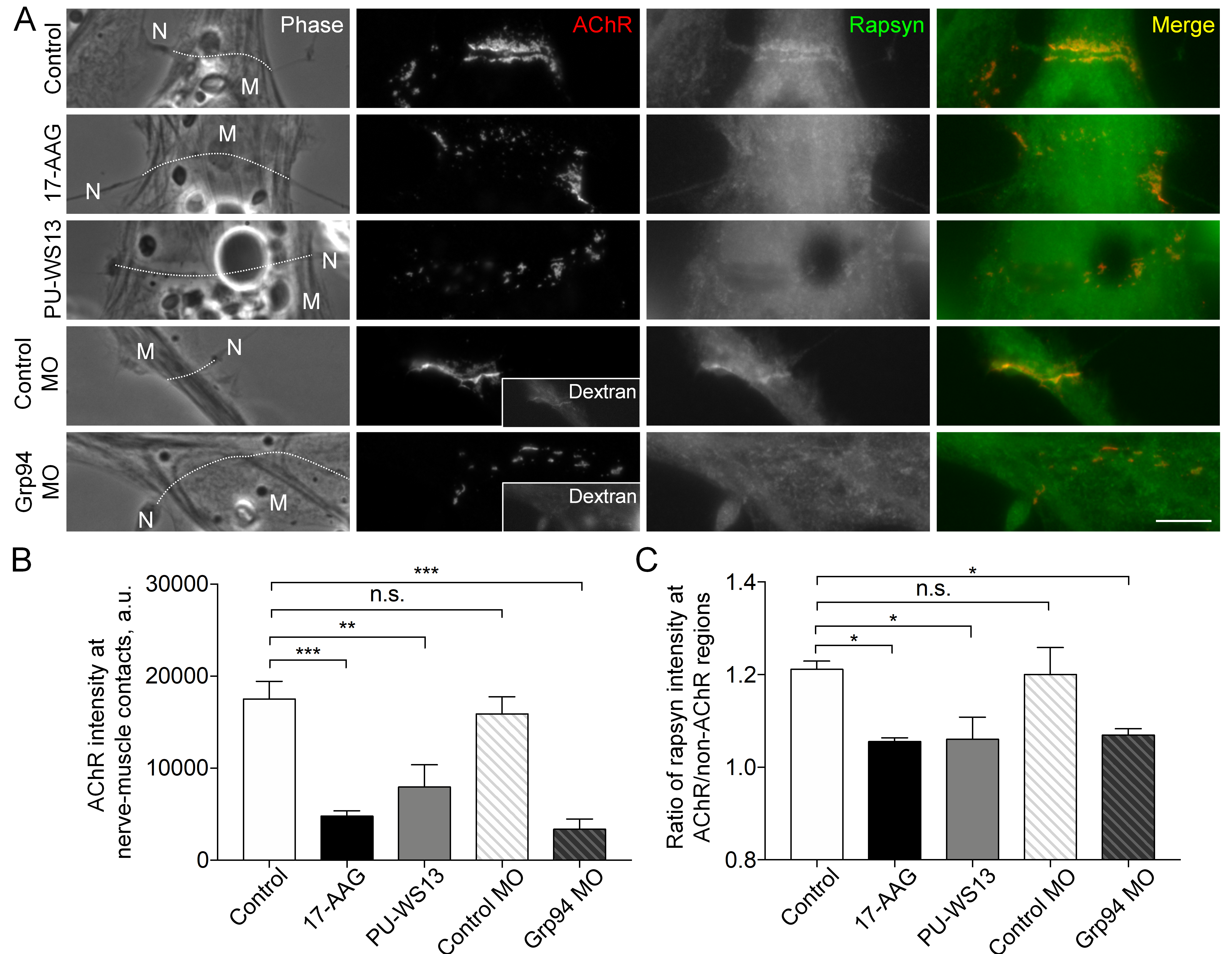

Supplement: Extended Data Figure 5-1 — HSP90 inhibition or Grp94 knock-down suppresses nerve-induced synaptic AChR clusters with reduced rapsyn localization. A, Representative images showing the effects of HSP90 inhibition or muscle Grp94 knock-down on nerve-induced AChR clustering and rapsyn localization at nerve-muscle contact sites. Dotted lines indicate nerve-muscle contacts. Insets show fluorescent dextran signals as cell-lineage tracer. “M”: muscle; “N”: neuron. B, C, Quantifications showing the fluorescence intensity of synaptic AChR clusters (B) and rapsyn (C) along the nerve-muscle contacts in 1-d-old Xenopus nerve-muscle co-cultures in the presence or absence of 17-AAG or PU-WS13 and in the chimeric co-cultures of WT neurons and muscle cells with Control MO or Grp94 MO; n = 26 (Control), n = 9 (17-AAG), n = 10 (PU-WS13), n = 18 (Control MO), and n = 14 (Grp94 MO) from four independent experiments. Scale bar: 10 μm. Data are shown as mean ± SEM. One-way ANOVA with Dunnett’s multiple comparisons test. *, **, and *** represent p ≤ 0.05, 0.01, and 0.001, respectively. n.s.: non-significant. Download Figure 5-1, TIF file. [file enu-eN-CFN-0025-20-s09.tif]

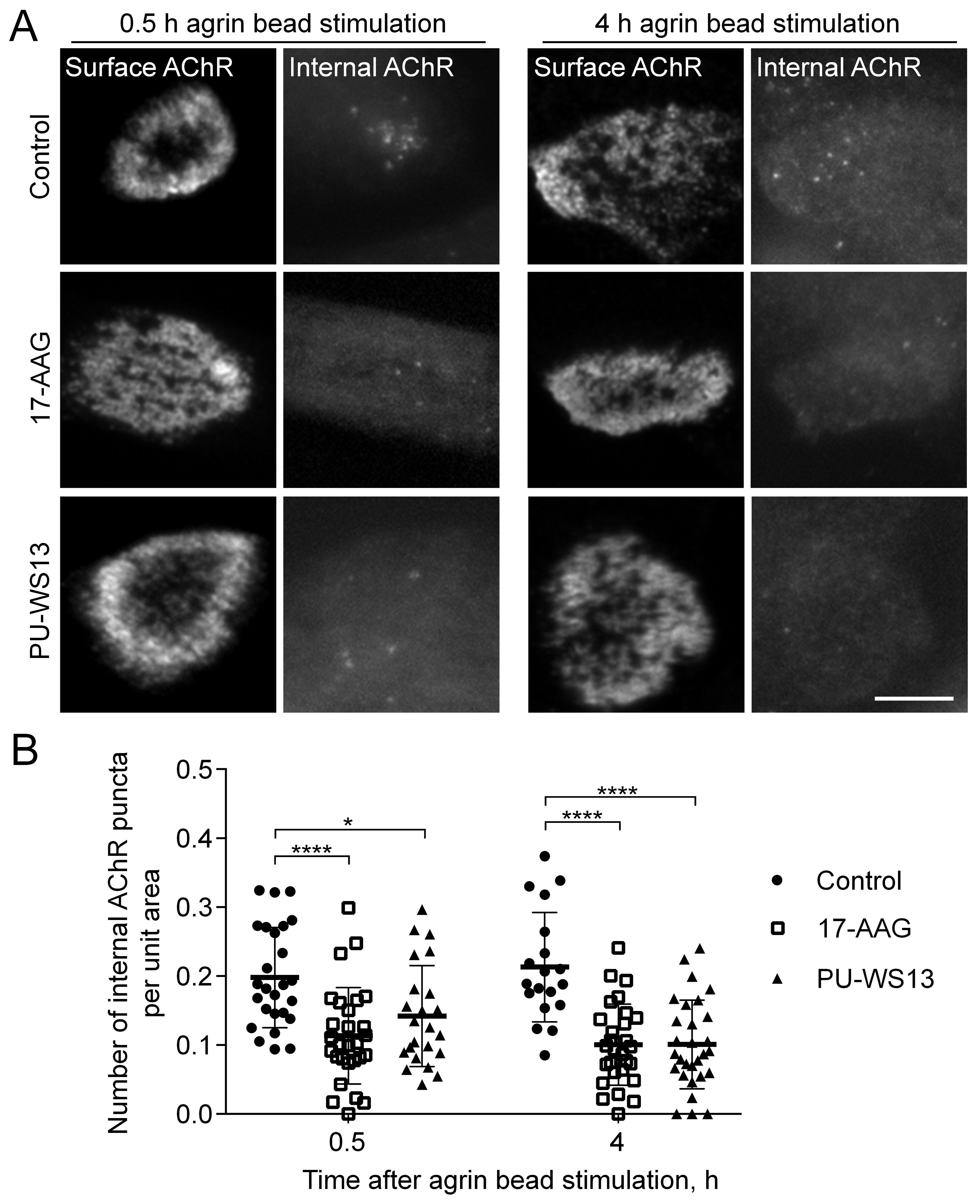

Supplement: Extended Data Figure 5-2 — Grp94 inhibition reduces the amount of AChR vesicles at aneural clusters in agrin-stimulated muscle cells. A, Representative images showing the effects of 17-AAG or PU-WS13 on AChR internalization at aneural clusters upon agrin stimulation. Images of aneural AChR clusters were taken from a single focal plane (surface AChR), while the maximal projection of intracellular AChR signals was constructed of a stack of 11 images at 0.2 μm per frame (internal AChR). B, Quantification showing the effects of 17-AAG or PU-WS13 on AChR internalization at aneural clusters upon agrin stimulation for 0.5 or 4 h; n = 27 (Control, 0.5 h), n = 28 (17-AAG, 0.5 h), n = 23 (PU-WS13, 0.5 h), n = 19 (Control, 4 h), n = 28 (17-AAG, 4 h), and n = 29 (PU-WS13, 4 h) muscle cells from three independent experiments. Scale bar: 5 μm. Data are shown as mean ± SD. One-way ANOVA with Dunnett’s multiple comparisons test. * and **** represent p ≤ 0.05 and 0.0001, respectively. Download Figure 5-2, TIF file. [file enu-eN-CFN-0025-20-s10.tif]
