# Supplementary material for: Grp94 Regulates the Recruitment of Aneural AChR Clusters for the Assembly of Postsynaptic Specializations by Modulating ADF/Cofilin Activity and Turnover
Source: eNeuro. 2020 Sep 2;7(5):ENEURO.0025-20.2020. doi: 10.1523/ENEURO.0025-20.2020 (PMC7540925; doi:10.1523/ENEURO.0025-20.2020)
Supplement: Extended Data Table 1-1 — A list of p values in comparing the relative amount of polar metabolites between control and 17-AAG-treated muscle cells. Download Table 1-1, DOCX file. [file enu-eN-CFN-0025-20-s03.docx]

| Polar metabolites | |
| --- | --- |
| Name | *p*-value |
| Pyruvic acid | 1 |
| Xylose | 0.73312 |
| Arabinose | 0.55341 |
| Ribose | 0.37583 |
| Lactic acid | 0.48562 |
| Alanine | 0.55433 |
| Mannose | 1 |
| Fructose | 0.09063 |
| 3-Hydroxybutyric acid | 0.98647 |
| Galactose | 0.65791 |
| Histidine | 0.82478 |
| Valine | 0.37583 |
| KMV | 1 |
| 2-Hydroxyglutaric acid | 0.81366 |
| Leucine | 0.84556 |
| Glycerol | 0.42265 |
| Succinic acid | 0.55036 |
| Proline | 0.68687 |
| Urea | 0.80296 |
| Glycine | 1 |
| Benzoic acid | 0.63306 |
| Isoleucine | 0.29524 |
| Cholesterol | 0.82478 |
| Fumaric acid | 0.71219 |
| Serine | 1 |
| Threonine | 0.37583 |
| Oxalacetic acid | 1 |
| Malic acid | 0.48917 |
| Aspartic acid | 0.64283 |
| Methionine | 1 |
| Gamma-aminobutyric acid | 1 |
| Phosphoenolpyruvic acid | 1 |
| Alpha-ketoglutaric acid | 0.64283 |
| Glutamic acid | 1 |
| Phenylalanine | 0.87856 |
| Asparagine | 1 |
| Dihydroxyacetone phosphate | 0.52331 |
| Glutamine | 1 |
| Glycerol 1-phosphate | 1 |
| Isocitric acid | 0.91967 |
| Citric acid | 1 |
| 3-Phosphoglyceric acid | 1 |
| 3,4-Dihydroxyphenylacetic acid | 1 |
| Glucose | 0.74394 |
| Lysine | 1 |
| Adrenaline | 0.98438 |
| Tyrosine | 1 |
| Ribose-5-phosphate | 1 |
| Dopamine | 0.42265 |
| Tryptophan | 1 |
| Glucose-6-phosphate | 1 |
| Cystine | 0.25034 |
| 6-Phosphogluconic acid | 0.68335 |
| Fructose-6-phosphate | 1 |
| Serotonin | 1 |
| Fructose-1,6-diphosphate | 0.60728 |
