# Supplementary material for: Grp94 Regulates the Recruitment of Aneural AChR Clusters for the Assembly of Postsynaptic Specializations by Modulating ADF/Cofilin Activity and Turnover
Source: eNeuro. 2020 Sep 2;7(5):ENEURO.0025-20.2020. doi: 10.1523/ENEURO.0025-20.2020 (PMC7540925; doi:10.1523/ENEURO.0025-20.2020)
Supplement: Extended Data Table 1-2 — A list of p values in comparing the relative amount of fatty acids between control and 17-AAG-treated muscle cells. Download Table 1-2, DOCX file. [file enu-eN-CFN-0025-20-s04.docx]

| Fatty acids | |
| --- | --- |
| Name | *p*-value |
| C6:0 | 1 |
| C14:0 | 0.4795 |
| C14:1 | 0.9295 |
| C15:0 | 1 |
| C17:0 | 1 |
| C18:0 | 0.42265 |
| C18:1, trans | 0.81366 |
| C18:2, trans | 0.97293 |
| C18:3 n6 | 0.50018 |
| C18:3 n3 | 0.42265 |
| C20:0 | 1 |
| C20:1 n9 | 1 |
| C20:2 | 1 |
| C21:0 | 1 |
| C20:3 n6 | 1 |
| C20:4 n6 | 0.42265 |
| C20:3 n3 | 0.6531 |
| C22:0 | 1 |
| C22:1 | 0.94006 |
| C22:2 | 1 |
| C23:0 | 0.97424 |
| C24:0 | 1 |
| C24:1 | 0.82478 |
| C26:0 | 1 |
|  |  |
